# Genetic Analysis of Chinese Cabbage Reveals Correlation Between Rosette Leaf and Leafy Head Variation

**DOI:** 10.3389/fpls.2018.01455

**Published:** 2018-10-04

**Authors:** XiaoXue Sun, Shuangxia Luo, Lei Luo, Xing Wang, Xueping Chen, Yin Lu, Shuxing Shen, Jianjun Zhao, Guusje Bonnema

**Affiliations:** ^1^Key Laboratory of Vegetable Germplasm Innovation and Utilization of Hebei, Collaborative Innovation Center of Vegetable Industry in Hebei, Department of Horticulture, Hebei Agricultural University, Baoding, China; ^2^Plant Breeding, Wageningen University & Research, Wageningen, Netherlands

**Keywords:** Chinese cabbage, rosette leaf, leafy head, heading degree, correlation, co-location of QTLs

## Abstract

To understand the genetic regulation of the domestication trait leafy-head formation of Chinese cabbages, we exploit the diversity within *Brassica rapa*. To improve our understanding of the relationship between variation in rosette-leaves and leafy heads, we phenotyped a diversity set of 152 Chinese cabbages. This showed correlation between rosette-leaf traits and both head traits and heading capacity. Interestingly, the leaf number of the mature head is not correlated to heading degree nor head shape. We then chose a non-heading pak choi genotype to cross to a Chinese cabbage to generate populations segregating for the leafy head traits. Both a large F2 (485 plants) and a smaller Doubled Haploid (88 lines) mapping population were generated. A high density DH-88 genetic map using the *Brassica* SNP array and an F2 map with a subset of these SNPs and InDel markers was used for quantitative trait locus (QTL) analysis. Thirty-one quantitative trait loci (QTLs) were identified for phenotypes of rosette-leaves in time and both heading degree and several heading traits. On chromosome A06 in both DH-88 and F2-485 QTLs for rosette leaf length and petiole length at different developmental days and an F2 QTL for head height co-located. Variation in head height, width and weight all correlate with variation in heading degree with co-locating QTLs, respectively, on chromosome A03, A05, and A08 in F2-485. The correlation between rosette-leaf and heading traits provides not only insight in the leaf requirements to form a head, but also can be used for selection by Chinese cabbage breeders.

## Introduction

*Brassica rapa* is an economical important species that includes many morphotypes that are cultivated as vegetable, oil and fodder crops (Prakash and Hinata, 1980; Gómez-Campo, 1999; Hanelt, 2001). It contributes many important leafy vegetable crops grown worldwide, with main production and consumption area’s in Asia and Europe (Dixon, 2007; Ge et al., 2011). The two most important *B. rapa* leafy morphotypes are Chinese cabbage with leafy heads and pak choi’s with flat smooth leaves and fleshy petioles that do not form a head. The general hypothesis is that Chinese cabbages have been domesticated from non-heading leafy pak choi’s in China (Li, 1984). Compared to the non-heading accessions, the heading types can be transported easily, stored long time and have better cold tolerance and higher yield (Daly and Tomkins, 1997).

Chinese cabbages go through four stages of vegetative growth: seedling, rosette, folding and heading stage (Ge et al., 2011). During seedling stage the leaves are round and have long petioles. Rosette leaves (RL) differentiate at the rosette stage and become large and round, with short petioles. At the end of the rosette stage, the folding leaves (FL) are also large and round with short petioles, but curve more inward and thereby form a mold for further head development. Unlike species that do not form a leafy head, the rosette and FL in heading plants remain metabolically active during the entire head development in order to supply the head with the products by photosynthesis (Yu et al., 2000; Wang et al., 2012). Leafy heads of Chinese cabbage are formed following the rosette and folding stages, when heading leaves develop an extreme upward and inward curvature giving rise to the leafy head that in effect becomes a nutrient storage organ. However, details of leaf development are rarely described and the correlation between variation of leaves in rosette/folding stage with timing of head formation and head shape is still unclear. As far as we know, only few genetic studies have been conducted on variation in rosette leaf traits and the relationship with head formation. Mao et al. (2014) phenotyped the RL and head shape at the heading stage and found a correlation between their morphology. Generally round heads had flat RL, cylindrical heads had RL with wavy margins and cone-like heads had RL that curved inward. The same team also recently published about the effects of microRNAs on leaf curvature, which is also highly relevant to understand leafy head formation in Chinese cabbage (Wang et al., 2014). In addition, several genomic studies are conducted that describe gene expression profiles during Chinese cabbage development (Wang et al., 2012, 2013; Gau et al., 2017). When these are combined with phenotyping of both the complete plant and the anatomy of leaves and meristems, our understanding of leafy head formation will increase.

Head formation is a quantitative trait, controlled by multiple genes, however, genetic mechanism of head formation is still unclear. Currently, few studies have identified quantitative trait loci (QTLs) for heading traits in Chinese cabbage, such as head weight, head diameter, head height and number of leaves. Kubo et al. (2010) constructed a map based on a mapping population consisting of 188 F2 plants derived from a cross between a Chinese cabbage and a vegetable turnip. For head formation, two QTLs were detected in both 2005 and 2007, with no common head formation QTLs detected. Ge et al. (2011) phenotyped 154 F3 families and identified twenty-seven QTL distributed over all nine linkage groups for gross weight, head weight, head length, head width, numbers of wrapper leaves and head forming leaves. Inoue et al. (2015) evaluated eight traits over 2 years (2010 and 2011) for a F2 population of 188 plants from a cross between two Chinese cabbage cultivars. They reported QTL for four heading traits (degree of head-top-leaf overlap, head height, head weight and the ratio of head height to head diameter) on linkage groups A1, A5, A7, and A8 in both years. Xiao et al. (2013) used a genetical genomics approach to study variation in leaf shape and size in *B. rapa*. For this purpose they used a DH population derived from a cross between non-heading pak choi and an oil type, yellow sarson. They identified several candidate genes for leaf variation and also revealed correlations between leaf traits and both plant architecture and flowering time.

In this paper we aimed to understand further the complexity of head formation during Chinese cabbage development. To achieve this goal, we performed the first global analysis of the correlation between leaf traits during development and head formation at heading stage using 152 Chinese cabbage cultivars. The data will substantially increase knowledge of Chinese cabbage heading related traits during leafy head development and provide research directions for QTL studies of heading related traits. We also generated an F1 population derived from a cross between heading Chinese cabbage (CC-48) and non-heading pak choi (PC-101). A doubled haploid population (DH-88) and an F2 population (F2-485) were developed from F1 by microspore culture and self-pollination, respectively. DH-88 and F2-485 linkage maps were constructed using 66 DH-88 and 485 F2-485 individuals for QTL analysis to detect loci related to RL related traits at rosette stage and leafy head formation at heading stage.

## Materials and Methods

### Plant Materials

#### Chinese Cabbage Genotypes for Morphological Traits Analysis

A collection of 152 *B. rapa L.* ssp. *pekinensis* (Chinese cabbage) accessions from different locations of China was used in this study (**Supplementary Table S1**). Among them, 69 accessions were inbred lines and 83 accessions were hybrid varieties (F1). Plants were grown in a random block design in the field in Hebei province in China, September 2013. Three blocks with fifteen plants of each accession were used for collection of morphological trait data.

#### DH-88 and F2-485 Mapping Populations

**FIGURE 1:**
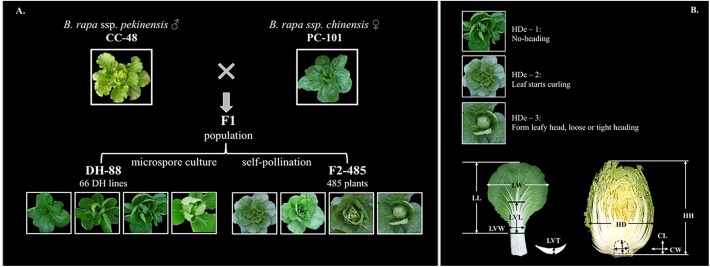
The strategy for constructing the Chinese cabbage (DH-88 and F2-485) populations **(A)** and description of the measurements of leaf and heading traits **(B)**. Head degree (HDe), head diameter (HD), head height (HH), core length (CL) and core width (CW) were recorded for the leafy heads. Leaf length (LL), leaf width (LW), leaf mid vein length (LVL), leaf mid vein width (LVW) and leaf mid vein thickness (LVT) were recorded for leaves.

The mapping populations used in this study consisted of a doubled haploid population (DH-88) and an F2 population (F2-485) (**Figure 1A**). The DH-88 and F2-485 have the same father line, heading Chinese cabbage CC-48 (*B. rapa* ssp. *pekinensis*: CGN06867) and mother line, non-heading pak choi PC-101 (*B. rapa* ssp. *chinensis*: CGN13926). The F1 was obtained from a cross between CC-48 and PC-101. The 66 lines of DH-88 were obtained by F1 plant microspore culture. From this F1, 485 F2 plants were produced after selfing.

For QTL analyses, the experiments were conducted in Hebei Agriculture University, Hebei, China. Sixty-six DH-88 lines, plus their parental lines and their F1’s were sown in seeding soil in the greenhouse, transplanted September 2014 to an open field at the Hebei Agriculture University, and grown under short day conditions (11 h photoperiod) until December 2014. The 485 F2 seeds were sown on the same experimental field September 2015, and grown under natural conditions until November 2015.

### Trait Evaluations and Phenotypic Data Analyses

The collection of 152 Chinese cabbage accessions were evaluated for 17 agronomic traits during heading stage and the traits scored in each individual plant are listed in **Table 1**. Five plants as biological replicates for each accession were evaluated for their agronomic traits.

**Table 1:** List of 17 evaluated phenotypic traits of 152 Chinese cabbage accessions, their ranges and descriptive statistics.

Traits	Description	Range	Mean	*SD^1^*	CV^2^
HDe	Head Degree: *Degree of heading leaf curvature at the time of harvest*	1–3	2.88	0.37	0.13
HtO	Head top Overlap: *Overlap degree of the top of heading leaf*	1–4	2.01	1.30	0.65
PL (cm)	Plant Length: *Length of the plant at the time of harvest*	18.5–75.0	39.84	9.37	0.24
PD (cm)	Plant Diameter: *Width of the plant at the time of harvest*	29.0–92.0	58.44	11.67	0.20
PW (kg)	Plant Weight: *Weight of the plant at the time of harvest*	0.37–7.69	2.96	1.25	0.42
LL (cm)	Leaf Length: *Length of the rosette leaf blade*	24.0–81.0	42.80	8.21	0.19
LW (cm)	Leaf Width: *Width of the rosette leaf*	16.0–42.0	27.51	5.23	0.19
LVW (cm)	Leaf mid Vein Width: *Width of the rosette leaf mid vein at the widest point*	3.5–28.0	6.94	2.40	0.35
LVL (cm)	Leaf mid Vein Length: *Length of the rosette leaf mid vein at the longest point*	10.3–60.0	23.81	6.59	0.28
LVT (cm)	Leaf mid Vein Thickness: *Width of the rosette leaf mid vein at the thickest point*	0.57–2.4	1.14	0.32	0.28
HD (cm)	Head Diameter: *Width of the leafy head without external leaves*	8.0–23.5	14.26	3.02	0.21
HH (cm)	Head Height: *Length of the leafy head without external leaves*	12.0–65.0	30.69	9.56	0.32
HW (kg)	Head Weight: *Weight of the leafy head without external leaves*	0.22–4.52	1.88	0.76	0.40
CW (cm)	Core Width: *Width of the core of leafy head*	1.6–6.0	3.75	0.85	0.23
CL (cm)	Core Length: *Length of the core of leafy head*	1.65–11.0	5.02	2.11	0.42
HI (ratio)	Head Index: *The ratio of leafy head length and leafy head width*	0.85–5.7	2.27	0.95	0.41
HLN	Head Leaf Number *Extant number of leaves of leafy head without external leaves ( > 2cm)*	11–69	33.16	9.98	0.30


*^1^Std. Deviation; ^2^Coefficient of Variation (CV) = (Std. Deviation / Mean) ^∗^ 100%.*

For QTL analysis, four traits of DH-88 and eight traits of the F2-485 population were evaluated at rosette and heading stages. In rosette stage, the Sixth rosette leaf was phenotyped for leaf width (LW), leaf length (LL), leaf petal length (PL) and leaf trichomes (LT) at 36, 41, 44, 47, 50, and 53 days after sowing for the DH-88 and 30, 34, 37, 41, 44, and 48 days after sowing for the F2-485 population. At the heading stage, the degree of heading (HDe) was classified on a 1–4 scale (1) non-heading, (2) leaf up curling, (3) loose heading, and (4) tight heading) by visual observation before maturity of the heads for both the DH-88 and F2-485 population (**Figure 1B**). At maturity, head diameter (HD), head height (HH), and head fresh weight (HW) were measured after discarding the loose outer leaves of all F2-485 plants at the same time. In DH-88 population, five biological replicates per DH line were used for traits observation.

The mean, standard deviation and variance were calculated for all traits by SPSS (Statistical Package for the Social Sciences, IBM) software. R package (“corrplot” package^1^) with Pearson correlation coefficient was used to calculate the correlation coefficients between traits and between populations.

### Genetic Map Construction and QTL Mapping

#### DNA Extraction

Genomic DNA was extracted from young leaves of DH-88 and F2-485 plants by the CTAB method (Murray and Thompson, 1980; Rogers and Bendich, 1985). Subsequently, the DNA was checked on gel, diluted to the same concentration with water and used to screen for SNP markers.

#### DH-88 and F2-485 Population Genetic Maps

TraitGenetics (Germany) developed a 60 K *B. napus* array. The parental genotypes CC-48 and PC-101 were screened for this array, which resulted in 5795 polymorphic SNPs, which were used to construct the genetic map. The DH-88 individuals were genotyped for a set of 5795 SNPs covering the 10 linkage groups. A subset of 899 specific SNPs was used for constructing the DH88 genetic linkage map (**Supplementary Table S2**) in this study. The F2-485 genetic map was based on 99 InDel markers and 36 SNP markers (**Supplementary Table S3**). The InDel markers were designed using the *B. rapa* data base^2^ and SNP markers were selected from the TraitGenetics set. SNP markers were used to anchor DH-88 and F2-485 populations. InDel markers were used for F2-485 genotyping by Native-PAGE method. SNP genotyping was carried out by high resolution melting analysis of small amplicons and HRMA was performed on 96-Well LightScanner System. Genetic maps for both populations were constructed by using JoinMap4.1 (Van Ooijen, 2006). After creating population nodes, LOD scores 8.0 to 10.0 were used to assign the markers into linkage groups (LGs) and Kosambi’s mapping function (Kosambi, 2016) was used to construct genetic maps. Consensus LGs of the two populations were constructed with common markers in both populations and drawn using Mapchart 2.3 (Voorrips, 2002).

#### DH-88 and F2-485 Population QTL Mapping of Heading Related Traits

Based on the genetic map, QTL mapping analysis was performed using MapQTL6 (Van Ooijen, 2009). QTL regions for testing traits were identified by interval mapping (IM) analysis. Based on the results of IM, multiple- QTL model mapping (MQM) was performed using the closest markers as cofactors. A QTL was considered significant when LOD thresholds were estimated with a genome-wide confidence level *p* < 0.005 (Doerge and Churchill, 1996).

## Results

### Diversity and Correlation Analyses of 17 Heading Related Traits Scored Over the 152 Chinese Cabbage Accessions

Phenotypic data for seventeen traits scored for the diversity set of 152 Chinese cabbages are shown in **Table 1**. All the quantitative traits showed normal distribution (**Supplementary Figure S1**). The coefficient of variation for “head-top-leaf overlap level” (HtO, 0.65) was higher than that of other traits, while coefficient of variation for “heading degree” (HDe, 0.13) was the lowest among the seventeen investigated traits.

**FIGURE 2:**
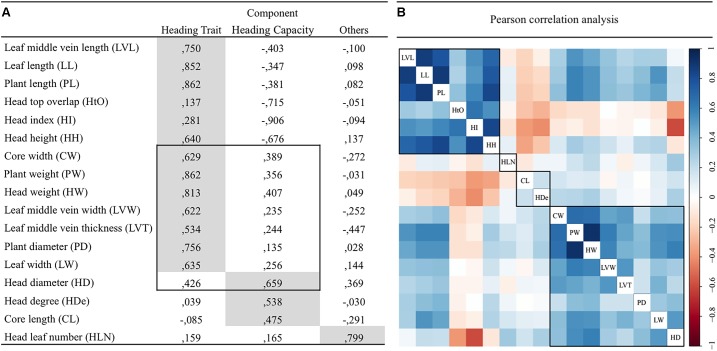
Principal component **(A)** and Pearson correlation **(B)** Pearson correlation analysis for 17 traits in 152 Chinese cabbage accessions.

The 17 traits were divided into three clusters based on the outcome of principal component analysis (**Figure 2A**). HDe, CL, HD were classified as “heading capacity traits” with regard to the Chinese cabbage head formation. Thirteen traits (LVL, LL, PL, HtO, HI, HH, CW, PW, HW, LVW, LVT, PD, and LW) were classified as “head type traits.” Based on the Eigen Value, CW, PW, HW, LVW, LVT, PD, LW traits mainly belong to the Head Trait component, but are also partly related to “Head capacity trait” component. This is also supported by Pearson correlation analysis (**Figure 2B**). Significant phenotypic correlations were found within the head type trait and within the heading capacity groups. CL and HDe clustered into one group and LVL, LL, PL, HtO, HI, HH formed another group, with significant correlation among these traits. The traits shared between Head Trait components and Heading capacity component grouped together based on their correlation rate. Only head leaf number (HLN) was negatively correlated with other traits in both component and correlation analysis.

### QTL Analysis in DH-88 and F2-485

#### Linkage Map Construction

##### Construction of DH-88 linkage maps

The 5795 SNPs were profiled over the DH-88 population, which resulted in 899 bins distributed over ten LGs spanning a total genetic distance of 1469.5 cM and average distance between markers is 0.92 cM. The LGs correspond to the Chinese cabbage reference map. We only found one inversion of adjacent SNP bins located at the bottom of A10 and few inconsistent bins located in chromosomes A02 and A07, respectively, compared to the Chiifu Chinese cabbage reference genome (**Supplementary Figure S2**).

##### Construction of F2-485 linkage map

The DH-88 population consists of only 66 DH lines, limiting mapping precision and detection of significant QTLs. In order to identify significant QTLs, a large F2-485 population with 485 F2 plants was generated and a linkage map was constructed. A variety of different types of molecular markers were used for the construction of this linkage map. These included 36 SNPs and 99 InDel markers distributed over the ten LGs spanning a genetic distance of 1145.7 cM with an average distance of 8.93 cM between markers (**Supplementary Figure S3**). In general, marker order was in agreement with the Chinese cabbage genome sequence v1.5^2^ with few exceptions. There were 36 common SNPs markers between DH-88 and F2-485 linkage maps (**Supplementary Table S4**). Common markers assist in comparison of the QTL positions on the two different linkage maps.

#### Correlation Analysis of Rosette Leaf and Heading Traits in Both DH-88 and F2-485

##### Development of RL in DH-88 and F2-485

**FIGURE 3:**
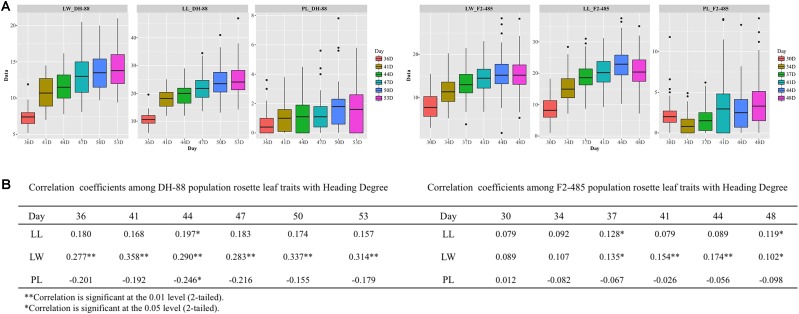
Sixth rosette leaf traits analysis in DH-88 and F2-485 populations through development. **(A)** Box plots of the rosette leaf length (LL), leaf width (LW), petiole length (PL), grouped by growth days (the days after sowing: DAS). **(B)** Correlation analysis between rosette leaf traits and “Final heading degree.”

Rosette leaves were phenotyped six times during their development; rosette leaf length and width continuously increase throughout the development (**Figure 3A**). In DH-88, LL increased significantly between 36DAS (days after sowing) and 47DAS, but growth ceased 50DAS as there was no significantly increase between 50DAS and 53DAS (*P* = 0.146). For LW, growth ceased after 47DAS (*P* = 0.167). In F2-485, LL increased significantly during all time points measured, while for LW no significant difference was seen between 44DAS and 48DAS (*P* = 0.763), and we can conclude that leaf growth ceased at 44DAS.

Correlations between LL, LW, and PL at different DAS with the trait “Final heading degree” are shown in **Figure 3B**. In both DH-88 and the F2-485 population, LL and PL are not significantly correlated with heading degree. LW however, is positively correlated with “Final heading degree” in both DH-88 and F2-485. In DH-88, the correlation between LW and heading degree was clear from 36DAS to 53DAS, while this correlation was only significant at 37DAS in F2-485.

##### Head trait analysis in F2 population

At the heading stage, the heading degree (HDe) was evaluated for 66 DH-88 lines and 485 F2 plants, while HH, HW, and HWe were only evaluated in the F2-485 population (**Supplementary Table S5**). In the F2 population, the variance was higher for HH than for HW and HWe. The correlation coefficient analyses showed that HH, HW, HWe, and HD were significantly correlated with each other. Correlation between HH and HD was lower, and not significant at the 0.01 level.

#### Detection of QTLs

Quantitative trait locus analyses for both leaf traits at the rosette stage and heading traits were performed for the DH-88 and F2-485 populations (**Table 2**).

**Table 2:** Quantitative trait loci found in DH-88 and F2-485 population.

QTL	StaAe	LG	Position (cM)	Nearest markers	LOD	R2 (%)
R_DHLL1A6	41D	A06	46.907–49.574	Bn-A06-p3934890(46.907)-Bn-A06-p4623401(49.574)	3.14	28.0
R_F2LL2A6	37D	A06	56.150	Bn-A06-p4452778(46.310)-A06-22-2(57.157)	4.48	8.9
R_F2LL3A6	41D	A06	55.150	Bn-A06-p4452778(46.310)-A06-22-2(57.157)	7.08	13.7
R_F2LL4A6	44D	A06	49.310	Bn-A06-p4452778(46.310)-A06-4(52.150)	9.93	18.7
R_F2LL5A6	48D	A06	55.150	A06-4(52.150)-A06-22-2(57.157)	9.65	18.3
R_DHLW1A3	47D	A03	84.414–89.414	Bn-A03-p14684924(84.414)-Bn-A03-p15627698(88.414)	3.87	33.3
R_F2LW2A4	44D	A04	11.211	A04-17-2(8.211)-A04-6(12.356)	3.38	6.8
R_DHPL1A6	36D	A06	53.349	Bn-A06-pp4056446(44.241)-Bn-A06-p5845542(53.682)	3.35	29.6
R_DHPL2A6	41D	A06	43.537	Bn-A06-p3496759(41.537)-Bn-A06-p3986320(45.574)	3.75	32.4
R_DHPL4A6	44D	A06	44.241	Bn-A06-p4056446	4.42	37.0
R_DHPL6A6	47D	A06	43.537	Bn-A06-p3496759(41.537)-Bn-A06-p3986320(45.574)	3.72	32.2
R_DHPL7A6	50D	A06	46.574	Bn-A06-p4056446(44.241)-Bn-A06-p4623401(49.574)	3.35	29.5
R_DHPL8A6	53D	A06	48.241	Bn-A06-p4021930	3.04	27.3
R_F2PL9A6	37D	A06	49.310	Bn-A06-p4452778(46.310)-A06-4(52.150)	8.14	15.5
R_F2PL10A6	41D	A06	49.310	Bn-A06-p4452778(46.310)-A06-4(52.150)	10.06	19
R_F2PL11A6	44D	A06	44.394	Bn-A06-p4452778(46.310)	5.97	11.6
R_F2PL12A6	48D	A06	49.310	Bn-A06-p4452778(46.310)-A06-4(52.150)	5.82	11.5
R_DHLT1A6	–	A06	147.610	Bn-A06-p21896359(147.610)	9.75	64
R_F2LT2A6	–	A06	127.7–132.9	Bn-A06-p22910339(106.266)	9.1	57.8
H_F2HH1A6	Head	A06	32.085	A06-43-1(31.085)-zyj6-6-2(59.563)	9.74	9.1
H_F2HH2A3	Head	A03	16.071	B03-11-1(10.071)-B03-12-2(19.627)	6.41	6.1
H_F2HW1A5	Head	A05	60.708	A05-7(60.708)	5.76	5.6
H_F2HWe1A8	Head	A08	10.000	A08-1-1(0.000)-Zyj8-2-2(10.978)	6.49	6.0
H_F2HWe2A6	Head	A06	62.563	Zyj6-6-2(59.563)-Zyj6-10-2(75.643)	5.89	5.5
H_DHHDe1A3	Head	A03	73.394	Bn-A03-p12863642(72.394)-Bn-A03-p12903591(73.728)	4.09	34.8
H_DHHDe2A8	Head	A08	8.140	Bn-A08-p1847328	3.39	29.9
H_F2HDe3A3	Head	A03	8.000	zyj3-3-2(0.000)-Bn-A03-p5439400(10.071)	5.90	5.5
H_F2HDe4A4	Head	A04	25.311	Zjy4-7(17.311)-zyj4-9-2(28.172)	4.46	4.2
H_F2HDe5A5	Head	A05	57.664	B05-2-1(54.664)-zyj5-18(58.964)	4.28	4.0
H_F2HDe6A5	Head	A05	77.975	zyj5-20-2(71.975)-A05-16-2(78.111)	4.91	4.6
H_F2De7A8	Head	A08	7.000	A08-1-1(0.000)-zyj8-2-2(10.978)	8.54	7.8


In the rosette stage, QTLs for rosette leaf area length (LL), LW and petiole length (PL) were detected for both the DH-88 and F2-485 population. For LL one QTL was detected in DH-88 (R_DHLL1), while four QTLs (R_F2LL2, 3, 4, 5) were detected in the F2-485. R_F2LL4 and R_F2LL5 had high LOD scores (9.93 and 9.65), and explained around 18% of the phenotypic variation. There was one LW QTL detected on A03 in DH-88 that explained 33% of the phenotypic variation and one on A04 in the F2 that explained 6.8%. For PL, 6 QTLs were detected in DH-88 on A06. Four QTLs on A06 for PL in F2-485 were identified for different developmental days. The QTL region of A06 showed multiple QTL for LL and PL.

In the heading stage, two QTLs for head height (H_F2HH1 and HH2), one for head width (H_F2HW1) and two for head weight (H_F2HWe1 and H_F2HWe2) were detected in the F2-485. For the heading degree trait, a total of five QTL in four LGs in F2-485 and two on two LGs in DH-88 were detected. Of the QTL detected for the HDe trait, H_F2De7 showed a higher LOD (8.54) value on A08 in F2-485. We found that the HDe QTL region of A03 and A08 were detected in both DH-88 and F2-485, while the HDe QTL on A04 and A05 were only detected in the F2-485 population.

In both DH-88 and F2-485, a major QTL for LT was detected on A06, explaining 64 and 57.8% of the variation.

#### Co-localization of QTLs Between Rosette Leaf and Head Traits

**FIGURE 4:**
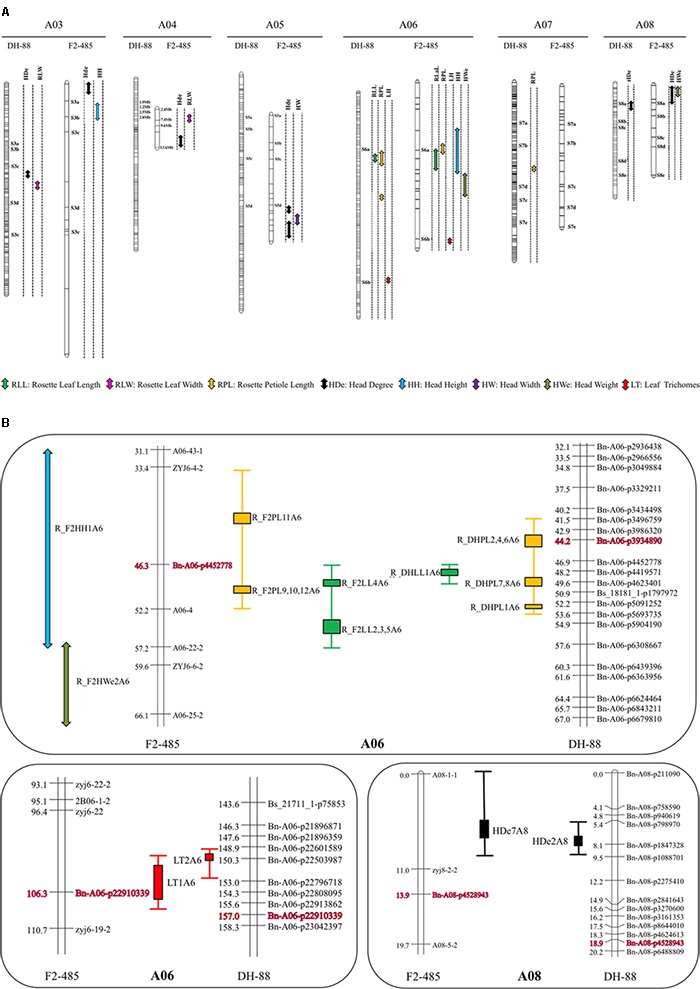
Quantitative trait loci hotspots for the DH-88 and F2-485 population. **(A)** QTLs are represented by colored vertical bars positioned at the right of each linkage group. Common markers between DH-88 and F2-485 are indicated by “Sxx” and marker information, which is also shown in **Supplementary Table S4**. **(B)** Zoom in of QTL hotspots of the rosette traits RLL (green), RPL (yellow), leaf trichomes LT (red) and heading degree HDe (black) on DH-88 and F2-485 on A06 and A08.

For the F2-485 population both rosette leaf and head traits were recorded, while for DH-88 population we measured rosette leaf traits and heading degree only. A total of 9 QTLs in rosette stage and 11 QTLs in heading stage were detected in the F2-485. Several QTLs were found to co-localize (**Figure 4A**). The QTL region of A03, A04, A05, A06, and A08 showed multiple QTLs for two till five traits. Head trait QTL H_F2HH2 and H_F2HDe3 were located at the top position of A03 (0.000–16.071 cM). Heading degree and rosette LW (H_F2HDe4 and R_F2LW2) were mapped closely linked on the top of LG A04. The bottom part of A05 (60.708–77.975 cM) showed overlapping QTL for head weight and heading degree traits: H_F2HW1 and H_F2HDe5, H_F2HDe6. QTL for both rosette leaf and head traits R_F2LL (2, 3, 4, 5), R_F2PL (9, 10, 11, 12), R_F2LT2, H_F2HH1 and H_F2HWe2 were clustered at the interval of 32.085–62.563 cM region on F2-485 LG A06. QTL for head traits H_F2HDe7 and H_F2We1 were detected at the top of A08 around 10.978 cM.

The positions of the rosette leaf RLL, RPL, LT, and heading degree (HDe) QTLs in this study were compared between DH-88 and F2-485 maps. A QTL for rosette leaf length (RLL: 46.8–49.5 cM) was mapped on DH88 LG A06. QTLs for RLL in F2 (46.3–57.2 cM) were detected on LG A06 below the common marker Bn-A06-p4452778. The SNP marker Bn-A06-p4452778 was linked with the QTL for RPL in both DH-88 (41.5–53.5 cM) and F2-485 (33.4–52.2 cM) in this study. QTLs for LT were mapped on LG A06 and a comparison of map positions could be made using the common Bn-A06-p22910339 marker in the maps. QTL for heading degree (HDe) in DH-88 (5.4–9.5 cM) and F2-485 (0.0–11.0 cM) on the top of LG A08 overlap, as they were linked to the common marker Bn-A08-p4528943 (**Figure 4B**).

## Discussion and Conclusion

Heading is an important breeding trait, as size and shape of the Chinese cabbage heads determine their market share. In addition, the heading trait is an important domestication trait that was domesticated around 1600 years ago (Tan, 1979; Li, 1981). Identification of QTL and the underlying causal genes can both shed light on the mutations and pathways that were selected for during domestication, but will also generate tools for breeders to select for optimal size and shape of Chinese cabbages.

### Head Formation Related Traits

So far, only few studies are published that study the genetics of leafy head formation (He et al., 2000; Wang et al., 2012; Yu et al., 2013), but only very few combined morphological variation at the rosette stage with variation in heading degree and shape. Mao et al. (2014) demonstrated that variation in expression of *TCP4* miRNA (*miRNA319*) explained head shape and the size, shape and curvature of RL. They showed clear correlation between morphology of leaves in rosette stage and final leafy head shape. In this study, we also extensively phenotyped leaf development during rosette stage and phenotyped the leafy head in a diverse collection of Chinese cabbage hybrids and inbred lines. This allowed us to evaluate variation and correlation between rosette and heading traits. We found that rosette leaf petiole- and leaf blade length were not correlated with final heading degree (HDe). However, rosette leaf width measured at several developmental stages was correlated with HDe during plant development in both F2-485 and DH88 populations. This correlation was weaker at early rosette stage and increased following the head formation process. The observation that rosette leaf width, which largely determines size and heading degree, are correlated fits with the physiology of Chinese cabbage. The special feature of heading Chinese cabbages is that younger leaves that initiate and grow inside the head are not photosynthetically active. As a consequence the outer leaves should remain active until harvest to generate photosynthetates and stimulate N uptake (Wang et al., 2012). Also, the redistribution from older outer leaves to the young inner leaves forming the head is of importance (Lammerts van Bueren and Struik, 2017). At later rosette stages when the head growth increases, the RL are thus very important and their width may affect the growth of the head.

A number of leafy head related traits were studied in the heading stage of the collection of Chinese cabbage accessions. By phenotype variation analysis, we found that in Chinese cabbage the variation in the extent to which the outer heading leaves overlap (HtO) was the largest, while the trait Heading Degree (HDe) showed little variation in the collection. We showed clear correlations among traits, with a cluster of traits related to heading capacity and a cluster of traits related to the shape of the head. The core of a Chinese cabbage head is the compact stem from which the leaves branch off. The shorter the core is, the denser the head. We detected strong correlation between core length (CL) and heading degree (HDe). Surprisingly, head leaf number (HLN) was not significantly correlated to the core length or to other head related traits. In this study the head weight was measured, however, head density (weight divided by volume) was not determined, a trait that is likely related to both leaf number and core length. Currently we developed tools to analyze 3D images that make it possible to calculate head shape, volume and density.

### Co-localized QTLs

By phenotyping a large F2 population derived from a cross between a PC and a CC parent for both rosette and heading traits and in addition phenotyping rosette traits in a DH population generated from the same cross, we found that QTLs for leaf size traits LL, LW, PL, the leaf trichome trait (LT) and leafy head traits HH, HW, HWe, HDe collocated in many cases. This was expected based on the correlations found between these traits.

For rosette leaves traits, common QTLs for leaf length (RLL), petiole length (RPL) and leaf trichome (LT) traits were identified on LG A06 in both the DH-88 and the F2-485 populations. However, several QTLs are found in a single population only. The two populations have very different sizes (485 F2 plants and 65 DH lines), which affects statistical power to detect QTL. In addition, F2 phenotypic data are based on single plants while DH data are based on several biological repeats of the same DH line. This, and the fact that all loci are homozygous in the DH, while in F2 plants homo- and heterozygotes both occur, and dominance relationships of the traits are generally unknown, can all affect QTL detection. QTLs for rosette leaf width (RLW) were identified on chromosome A03 in DH-88 and on chromosome A04 in F2-485. Very interestingly, the rosette leaf width (RLW) QTLs all co-located with heading degree (HDe) QTL. This as discussed based on their correlation, matches the physiological explanation that RL are the source of nutrients for the growing leafy head that serves as a sink. Colocation of QTL for RLL and RPL on LG A06 in both F2-485 and DH-88 population illustrates that total leaf length is correlated to petiole length.

For head related traits in the F2-485, we detected three QTLs for head length (HH) on LG A03, A06, A08, one QTL for head width (HW) on LG A05 and two QTLs for head weight (HWe) on LG A06, A08. Another QTL study in F3 families from a cross between small-and large heading Chinese cabbage lines identified QTLs for head related traits on the same LGs: HH on LG A03 and for HWe on LG A06 and A08 (Ge et al., 2011). In yet another study a head width QTL on LG A05 was detected (Yu et al., 2013). In the study of Yu et al. (2013) HW was phenotyped using 150 recombinant inbred lines from a cross between heading and non-heading Chinese cabbage in both 2011 and 2012 and one HW QTL on LG A05 was identified. In our study, we found that the head traits height, width and weight (HH, HW, and HWe) were highly correlated with heading degree (HDe) and their QTLs co-located on LG A03, LG A05 and LG A08. Very interestingly, colocation of QTL for RL traits (RLL, RPL) and head related traits (HH, HWe) were identified on LG A06 in F2-485. This again shows the relation between RL and leafy heads, where RL provide the nutrients for the growing leafy head.

In conclusion, we provide information on the relationship between leaf characteristics at rosette stage and leafy head formation. Colocation of QTL for heading related traits and for rosette leaf and heading traits suggests pleiotropic gene regulation of these complex traits. Knowledge about the relationship between the size and shape of RL that affect heading-degree, -size and -shape, is relevant for breeders that select for optimal quality of Chinese cabbage leafy heads.

## Author Contributions

XS and SL designed the experiment. XS performed the data analysis and contributed to manuscript writing. SL arranged the field experiments and collected phenotypic data. LL and XW did F2-485 genotyping. XC and YL helped in the discussion. SS and JZ provided funds for this study. JZ was involved in discussions. GB supervised the whole experiment and wrote the manuscript. All authors approved the final manuscript.

## Conflict of Interest Statement

The authors declare that the research was conducted in the absence of any commercial or financial relationships that could be construed as a potential conflict of interest.

## Footnotes

1 https://www.r-project.org

2 http://brassicadb.org/brad/
