# Supplementary material for: Genetic Analysis of Chinese Cabbage Reveals Correlation Between Rosette Leaf and Leafy Head Variation
Source: Front Plant Sci. 2018 Oct 4;9:1455. doi: 10.3389/fpls.2018.01455 (PMC6180146; doi:10.3389/fpls.2018.01455)
Supplement: Supplementary file 1 [file Table_1.docx]

**Supplemental tables and figures**

Table S1. 152 Chinese cabbage hybrids and inbred lines used for morphological trait analysis

| **No.** | **Accession type** | **No.** | **Accession type** | **No.** | **Accession type (name)** |
| --- | --- | --- | --- | --- | --- |
| H-1 | F_1_（H-113×H-139） | H-52 | F_1_（H-142×H-140） | H-127 | Inbred line |
| H-2 | F_1_（H-111×H-139） | H-53 | F_1_（H-142×H-144） | H-128 | Inbred line |
| H-3 | F_1_（H-139×H-111） | H-54 | F_1_（H-142×H-139） | H-129 | Inbred line |
| H-4 | F_1_（H-139×H-115） | H-55 | F_1_（H-111×H-139） | H-130 | Inbred line |
| H-5 | F_1_（H-112×H-111） | H-56 | F_1_（H-143×H-139） | H-131 | Inbred line |
| H-6 | F_1_（H-112×H-115） | H-57 | F_1_（H-139×H-140） | H-132 | Inbred line |
| H-7 | F_1_（H-112×H-115） | H-58 | F_1_（H-139×H-144） | H-133 | Inbred line |
| H-8 | F_1_（H-113×H-139） | H-59 | F_1_（H-182×H-140） | H-134 | Inbred line |
| H-9 | F_1_（H-158×H-153） | H-60 | F_1_（H-188×H-161） | H-135 | Inbred line |
| H-10 | F_1_（H-111×H-187） | H-61 | F_1_（H-152×H-183） | H-136 | Inbred line |
| H-11 | F_1_（H-149×H-150） | H-62 | F_1_（H-152×H-188） | H-137 | Inbred line |
| H-12 | F_1_（H-156×H-159） | H-63 | F_1_（H-181×H-147） | H-138 | Inbred line |
| H-13 | F_1_（H-157×H-155） | H-64 | F_1_（H-190×H-136） | H-139 | Inbred line |
| H-14 | F_1_（H-189×H-160） | H-65 | F_1_（H-184×H-136） | H-140 | Inbred line |
| H-15 | F_1_（H-133×H-134） | H-66 | F_1_（H-185×H-186） | H-141 | Inbred line |
| H-16 | F_1_（H-133×H-120） | H-67 | F_1_（H-185×H-148） | H-142 | Inbred line |
| H-17 | F_1_（H-134×H-131） | H-68 | F_1_（H-131×H-159） | H-143 | Inbred line |
| H-18 | F_1_（H-180×H-173） | H-69 | F_1_（H-181×H-115） | H-144 | Inbred line |
| H-19 | F_1_（H-180×H-129） | H-70 | F_1_（H-178×H-179） | H-145 | Inbred line |
| H-20 | F_1_（H-119×H-115） | H-71 | F_1_（H-176×H-177） | H-146 | Inbred line |
| H-21 | F_1_（H-119×H-139） | H-72 | F_1_（H-174×H-175） | H-147 | Inbred line |
| H-22 | F_1_（H-119×H-129） | H-73 | F_1_（H-169×H-170） | H-148 | Inbred line |
| H-23 | F_1_（H-121×H-134） | H-101 | F_1_（H-111×H-113） | H-149 | Inbred line |
| H-24 | F_1_（H-115×H-114） | H-102 | F_1_（H-189×H-161） | H-150 | Inbred line |
| H-25 | F_1_（H-115×H-114） | H-103 | F_1_（H-187×H-139） | H-151 | Inbred line |
| H-26 | F_1_（H-115×H-135） | H-104 | F_1_（H-129×H-140） | H-152 | Inbred line |
| H-27 | F_1_（H-124×H-125） | H-105 | F_1_（H-139×H-163） | H-153 | Inbred line |
| H-28 | F_1_（H-117×H-134） | H-106 | F_1_ | H-154 | Inbred line |
| H-29 | F_1_（H-135×H-116） | H-107 | F_1_ | H-155 | Inbred line |
| H-30 | F_1_（H-129×H-120） | H-108 | F_1_ | H-156 | Inbred line |
| H-31 | F_1_（H-129×H-123） | H-109 | F_1_ | H-157 | Inbred line |
| H-32 | F_1_（H-116×H-127） | H-110 | F_1_ | H-158 | Inbred line |
| H-33 | F_1_（H-114×H-173） |  |  | H-159 | Inbred line |
| H-34 | F_1_（H-114×H-115） | H-112 | Inbred line | H-160 | Inbred line |
| H-35 | F_1_（H-114×H-125） | H-113 | Inbred line | H-161 | Inbred line |
| H-36 | F_1_（H-114×H-127） | H-114 | Inbred line | H-162 | Inbred line |
| H-37 | F_1_（H-127×H-114） | H-115 | Inbred line | H-163 | Inbred line |
| H-38 | F_1_（H-114×H-139） | H-116 | Inbred line | H-164 | Inbred line |
| H-39 | F_1_（H-114×H-129） | H-117 | Inbred line | H-165 | Inbred line |
| H-40 | F_1_（H-130×H-131） | H-118 | Inbred line | H-166 | Inbred line |
| H-41 | F_1_（H-163×H-111） | H-119 | Inbred line | H-167 | Inbred line |
| H-42 | F_1_（H-139×H-181） | H-120 | Inbred line | H-168 | Inbred line |
| H-43 | F_1_（'H-139×H-133） | H-121 | Inbred line | H-169 | Inbred line |
| H-44 | F_1_（H-127×H-134） | H-122 | Inbred line | H-170 | Inbred line |
| H-45 | F_1_（H-127×H-115） | H-123 | Inbred line | H-171 | Inbred line |
| H-46 | F_1_（H-127×H-124） | H-124 | Inbred line | H-172 | Inbred line |
| H-47 | F_1_（H-127×H-125） | H-125 | Inbred line | H-173 | Inbred line |
| H-48 | F_1_（H-127×H-128） | H-126 | Inbred line | H-174 | Inbred line |
| H-49 | F_1_（H-127×H-129） | H-130 | Inbred line | H-175 | Inbred line |
| H-50 | F_1_（H-118×H-126） | H-131 | Inbred line | H-176 | Inbred line |
| H-51 | F1（H-118×H-139) | H-132 | Inbred line | H-177 | Inbred line |

Table S2. Marker names (899 SNPs) with their corresponding genes used in the DH-88 map

| **Marker** | **LG** | **Gene** | **Marker** | **LG** | **Gene** |
| --- | --- | --- | --- | --- | --- |
| Bn-A01-p80385 | A01 | Bra011885 | Bn-A01-p8043776 | A01 | Bra013844 |
| Bn-A01-p95718 | A01 | Bra011881 | Bn-A01-p8076080 | A01 | Bra013854 |
| Bs_19244_1-p698816 | A01 | Bra011778 | Bn-A01-p7850092 | A01 | Bra013806 |
| Bn-A01-p916774 | A01 | Bra011714 | Bn-A01-p9223420 | A01 | Bra013810 |
| Bn-A01-p820317 | A01 | Bra011736 | Bn-A01-p10000230 | A01 | Bra026326 |
| Bn-A01-p939202 | A01 | Bra011711 | Bn-A01-p10340159 | A01 | Bra026260 |
| Bn-A01-p994722 | A01 | Bra011699 | Bn-A01-p10299187 | A01 | Bra026266 |
| Bn-A01-p1012990 | A01 | Bra011696 | Bs_15803_1-p813514 | A01 | Bra026233 |
| Bn-A01-p1409723 | A01 | Bra011614 | Bn-A01-p10499882 | A01 | Bra026230 |
| Bn-A01-p1067371 | A01 | Bra011683 | Bn-A01-p11174997 | A01 | Bra038446 |
| Bn-A01-p1314440 | A01 | Bra011638 | Bn-A01-p11238778 | A01 | Bra033895 |
| Bn-A01-p1073387 | A01 | Bra011682 | Bn-A01-p12593173 | A01 | Bra036865 |
| Bn-A01-p1149698 | A01 | Bra011670 | Bn-A01-p11543126 | A01 | Bra033505 |
| Bn-A01-p1152359 | A01 | Bra011669 | Bn-A01-p11947255 | A01 | Bra039568 |
| Bn-A01-p1531829 | A01 | Bra011588 | Bn-A01-p14948614 | A01 | Bra029926 |
| Bn-A01-p1439621 | A01 | Bra011605 | Bn-A01-p16261297 | A01 | Bra021331 |
| Bn-A01-p1855903 | A01 | Bra011516 | Bn-A01-p19512499 | A01 | Bra023741 |
| Bn-A01-p1951988 | A01 | Bra011495 | Bn-A01-p19993388 | A01 | Bra023797 |
| Bn-A01-p1966955 | A01 | Bra011488 | Bs_21225_1-p53803 | A01 | Bra038246 |
| Bn-A01-p2111978 | A01 | Bra005478 | Bn-A01-p21444041 | A01 | Bra038229 |
| Bn-A01-p2148059 | A01 | Bra011444 | Bs_15794_2-p251230 | A01 | Bra037538 |
| Bs_15838_1-p2261053 | A01 | Bra011411 | Bn-A01-p22743403 | A01 | Bra021291 |
| Bn-A01-p2445468 | A01 | Bra011380 | Bn-A01-p22949106 | A01 | Bra021263 |
| Bn-A01-p2517658 | A01 | Bra011367 | Bn-A01-p23538267 | A01 | Bra021179 |
| Bn-A01-p3077309 | A01 | Bra011252 | Bn-A01-p23472646 | A01 | Bra021187 |
| Bn-A01-p3070657 | A01 | Bra011253 | Bn-A01-p23772904 | A01 | Bra021140 |
| Bn-A01-p3744726 | A01 | Bra011133 | Bn-A01-p23813837 | A01 | Bra021128 |
| Bn-A01-p4883188 | A01 | Bra013305 | Bs_27278_1-p1211 | A01 | Bra021552 |
| Bn-A01-p3829785 | A01 | Bra011113 | Bn-A01-p23842865 | A01 | Bra021121 |
| Bs_17827_1-p629351 | A01 | Bra013443 | Bn-A01-p24319792 | A01 | Bra021591 |
| Bn-A01-p5730640 | A01 | Bra012513 | Bs_22115_1-p155376 | A01 | Bra021583 |
| Bn-A01-p6349933 | A01 | Bra013560 | Bn-A01-p24756888 | A01 | Bra021524 |
| Bn-A01-p6376105 | A01 | Bra013568 | Bn-A01-p24848629 | A01 | Bra021514 |
| Bn-A01-p6482543 | A01 | Bra013585 | Bn-A01-p24862401 | A01 | Bra021512 |
| Bn-A01-p6877281 | A01 | Bra013652 | Bs_16567_1-p97437 | A01 | Bra021491 |
| Bn-A01-p7121832 | A01 | Bra013684 | Bn-A01-p26604864 | A01 | Bra038689 |
| Bn-A01-p7714470 | A01 | Bra013774 | Bn-A01-p26688333 | A01 | Bra038672 |
| Bn-A01-p27299339 | A01 | Bra034132 | Bn-A02-p20371730 | A02 | Bra026577 |
| Bn-A01-p27822000 | A01 | Bra040041 | Bn-A02-p19674831 | A02 | Bra024927 |
| Bn-A01-p27722989 | A01 | Bra040063 | Bn-A02-p19683093 | A02 | Bra022150 |
| Bn-A01-p27834452 | A01 | Bra040038 | Bn-A02-p19317643 | A02 | Bra022100 |
| Bn-A01-p27877194 | A01 | Bra040024 | Bn-A02-p17156661 | A02 | Bra033167 |
| Bn-A01-p28346931 | A01 | Bra040113 | Bn-A02-p19534217 | A02 | Bra003154 |
| Bn-A01-p25259103 | A01 | Bra021426 | Bn-A02-p19566951 | A02 | Bra037910 |
| Bn-A01-p25311527 | A01 | Bra001065 | Bn-A02-p16571709 | A02 | Bra008559 |
| Bs_15709_1-p74248 | A01 | Bra021418 | Bn-A02-p16259298 | A02 | Bra033147 |
| Bn-A01-p25061137 | A01 | Bra021477 | Bs_22749_1-p250319 | A02 | Bra027897 |
| Bn-A01-p25235998 | A01 | Bra021433 | Bn-A05-p9247748 | A02 | Bra018559 |
| Bn-A02-p25966536 | A02 | Bra029309 | Bn-A02-p16526928 | A02 | Bra033137 |
| Bn-A02-p26411585 | A02 | Bra029235 | Bn-A02-p15693192 | A02 | Bra008447 |
| Bn-A02-p26304354 | A02 | Bra029252 | Bn-A02-p15912199 | A02 | Bra008484 |
| Bn-A02-p26419529 | A02 | Bra035852 | Bn-A02-p14103759 | A02 | Bra008253 |
| Bs_16139_1-p655325 | A02 | Bra031863 | Bn-A02-p14208801 | A02 | Bra008265 |
| Bn-A02-p26421790 | A02 | Bra029233 | Bn-A02-p13874627 | A02 | Bra008216 |
| Bs_17177_1-p105819 | A02 | Bra029301 | Bn-A02-p13803435 | A02 | Bra023991 |
| Bn-A02-p25827208 | A02 | Bra029333 | Bn-A02-p12688495 | A02 | Bra008034 |
| Bn-A02-p25736307 | A02 | Bra029347 | Bn-A02-p12295281 | A02 | Bra007988 |
| Bn-A02-p25081911 | A02 | Bra020485 | Bn-A02-p12581342 | A02 | Bra008018 |
| Bn-A02-p25081916 | A02 | Bra020485 | Bn-A02-p13627045 | A02 | Bra008182 |
| Bn-A02-p25199281 | A02 | Bra020468 | Bn-A02-p11998921 | A02 | Bra007941 |
| Bn-A02-p25201372 | A02 | Bra020468 | Bn-A02-p11937221 | A02 | Bra007930 |
| Bn-A02-p25181474 | A02 | Bra020471 | Bn-A02-p11842725 | A02 | Bra007919 |
| Bn-A02-p24967958 | A02 | Bra020497 | Bn-A02-p11929538 | A02 | Bra007927 |
| Bs_17623_1-p120681 | A02 | Bra020537 | Bn-A02-p11817742 | A02 | Bra017390 |
| Bn-A02-p24449690 | A02 | Bra020582 | Bn-A02-p11355276 | A02 | Bra038308 |
| Bn-A02-p24533751 | A02 | Bra030412 | Bn-A02-p11039119 | A02 | Bra038353 |
| Bn-A02-p23889188 | A02 | Bra020658 | Bn-A02-p11160726 | A02 | Bra038340 |
| Bn-A02-p24415454 | A02 | Bra020589 | Bn-A02-p10409103 | A02 | Bra040734 |
| Bn-A02-p23856500 | A02 | Bra020666 | Bn-A02-p10949322 | A02 | Bra038367 |
| Bn-A02-p23488731 | A02 | Bra020727 | Bn-A02-p9480465 | A02 | Bra022487 |
| Bn-A02-p23523709 | A02 | Bra020719 | Bs_23813_1-p581761 | A02 | Bra022493 |
| Bn-A02-p21378450 | A02 | Bra017160 | Bn-A02-p9140734 | A02 | Bra022543 |
| Bn-A02-p21639869 | A02 | Bra033055 | Bn-A02-p7409699 | A02 | Bra035586 |
| Bn-A02-p21930156 | A02 | Bra033012 | Bn-A02-p7476709 | A02 | Bra035571 |
| Bn-A02-p22847472 | A02 | Bra038968 | Bn-A02-p7595911 | A02 | Bra035547 |
| Bn-A02-p20200723 | A02 | Bra026556 | Bs_21250_1-p212592 | A02 | Bra035594 |
| Bn-A02-p20201241 | A02 | Bra026556 | Bs_16804_1-p64080 | A02 | Bra020444 |
| Bn-A02-p20371774 | A02 | Bra026577 | Bn-A02-p6751482 | A02 | Bra007891 |
| Bn-A02-p7245763 | A02 | Bra035616 | Bn-A03-p4202242 | A03 | Bra006596 |
| Bs_16269_1-p197772 | A02 | Bra020402 | Bn-A03-p4408006 | A03 | Bra006637 |
| Bn-A02-p6478309 | A02 | Bra020381 | Bn-A03-p4382093 | A03 | Bra006633 |
| Bn-A02-p6476748 | A02 | Bra020380 | Bn-A03-p4997351 | A03 | Bra006772 |
| Bn-A02-p6149358 | A02 | Bra020310 | Bn-A03-p5532043 | A03 | Bra028942 |
| Bn-A02-p5644858 | A02 | Bra020221 | Bn-A03-p5273046 | A03 | Bra006835 |
| Bn-A02-p5592678 | A02 | Bra020213 | Bn-A03-p5439400 | A03 | Bra028925 |
| Bs_17522_1-p1818546 | A02 | Bra020140 | Bn-A03-p5770833 | A03 | Bra028994 |
| Bs_17522_1-p1837209 | A02 | Bra020140 | Bs_18322_1-p1368804 | A03 | Bra029108 |
| Bs_27670_1-p366718 | A02 | Bra023692 | Bn-A03-p6332797 | A03 | Bra029095 |
| Bn-A02-p4102727 | A02 | Bra023590 | Bn-A03-p5809786 | A03 | Bra006848 |
| Bs_15714_1-p642855 | A02 | Bra023585 | Bn-A03-p5784532 | A03 | Bra028996 |
| Bs_15714_1-p615978 | A02 | Bra023591 | Bn-A03-p5814447 | A03 | Bra029003 |
| Bn-A02-p3897546 | A02 | Bra023551 | Bn-A03-p7011698 | A03 | Bra022779 |
| Bn-A02-p3897213 | A02 | Bra023551 | Bs_21312_1-p1111796 | A03 | Bra022908 |
| Bn-A02-p4237047 | A02 | Bra023622 | Bn-A03-p6577213 | A03 | Bra029149 |
| Bn-A02-p3760247 | A02 | Bra023521 | Bn-A03-p7920650 | A03 | Bra022951 |
| Bs_15714_1-p977841 | A02 | Bra023533 | Bn-A03-p8308938 | A03 | Bra023030 |
| Bn-A02-p2568989 | A02 | Bra028684 | Bn-A03-p8398790 | A03 | Bra023047 |
| Bn-A02-p1344870 | A02 | Bra028623 | Bn-A03-p9429437 | A03 | Bra034939 |
| Bn-A02-p1327787 | A02 | Bra028619 | Bn-A03-p10047998 | A03 | Bra000229 |
| Bn-A02-p2364479 | A02 | Bra028726 | Bn-A03-p10011955 | A03 | Bra016935 |
| Bn-A02-p1997812 | A02 | Bra028796 | Bs_16002_1-p329748 | A03 | Bra000357 |
| Bn-A03-p135145 | A03 | Bra039049 | Bn-A03-p11294370 | A03 | Bra000478 |
| Bn-A03-p709332 | A03 | Bra005800 | Bn-A03-p11704711 | A03 | Bra000548 |
| Bn-A03-p1532114 | A03 | Bra005994 | Bn-A03-p12411362 | A03 | Bra000679 |
| Bn-A03-p1639446 | A03 | Bra006017 | Bn-A03-p12863642 | A03 | Bra000751 |
| Bn-A03-p1719791 | A03 | Bra021727 | Bn-A03-p13061709 | A03 | Bra000779 |
| Bn-A03-p1762980 | A03 | Bra006050 | Bn-A03-p13802162 | A03 | Bra000898 |
| Bn-A03-p1638326 | A03 | Bra006017 | Bn-A03-p14330609 | A03 | Bra001003 |
| Bn-A03-p2100226 | A03 | Bra006120 | Bn-A03-p14473296 | A03 | Bra001036 |
| Bn-A03-p2071494 | A03 | Bra006111 | Bn-A03-p14418650 | A03 | Bra035135 |
| Bn-A03-p2683534 | A03 | Bra006247 | Bn-A03-p14684924 | A03 | Bra001090 |
| Bn-A03-p2793288 | A03 | Bra006276 | Bn-A03-p15627698 | A03 | Bra001279 |
| Bn-A03-p2879773 | A03 | Bra006287 | Bn-A03-p15708192 | A03 | Bra029815 |
| Bn-A03-p2976496 | A03 | Bra006316 | Bn-A03-p16742011 | A03 | Bra001507 |
| Bn-A03-p3098147 | A03 | Bra006341 | Bn-A03-p16812953 | A03 | Bra001519 |
| Bs_15877_1-p184815 | A03 | Bra006379 | Bn-A03-p16955732 | A03 | Bra001546 |
| Bs_15877_1-p184866 | A03 | Bra006379 | Bn-A03-p17182494 | A03 | Bra001582 |
| Bn-A03-p3698620 | A03 | Bra006480 | Bn-A03-p18014273 | A03 | Bra001714 |
| Bn-A03-p3544165 | A03 | Bra006450 | Bn-A03-p17790084 | A03 | Bra001672 |
| Bs_17298_1-p2049303 | A03 | Bra001588 | Bn-A03-p28036367 | A03 | Bra024038 |
| Bn-A03-p16989353 | A03 | Bra001550 | Bn-A03-p28153736 | A03 | Bra024025 |
| Bn-A03-p18376493 | A03 | Bra001774 | Bn-A03-p28153797 | A03 | Bra024025 |
| Bn-A03-p19202899 | A03 | Bra020742 | Bn-A03-p28256169 | A03 | Bra024010 |
| Bn-A03-p19441620 | A03 | Bra001934 | Bn-A03-p28928570 | A03 | Bra037016 |
| Bn-A03-p19573902 | A03 | Bra001952 | Bn-A03-p28878501 | A03 | Bra037021 |
| Bn-A03-p19768447 | A03 | Bra013226 | Bn-A03-p28982546 | A03 | Bra037009 |
| Bn-A03-p21292773 | A03 | Bra012963 | Bn-A04-p783339 | A04 | Bra014463 |
| Bn-A03-p21507915 | A03 | Bra012920 | Bn-A04-p24411 | A04 | Bra040436 |
| Bn-A03-p20670561 | A03 | Bra013066 | Bn-A04-p761383 | A04 | Bra014459 |
| Bn-A03-p20786060 | A03 | Bra024350 | Bn-A04-p813173 | A04 | Bra014467 |
| Bn-A03-p20560569 | A03 | Bra013083 | Bn-A04-p1212589 | A04 | Bra007479 |
| Bn-A03-p22098429 | A03 | Bra012815 | Bn-A04-p1042273 | A04 | Bra014498 |
| Bn-A03-p21864294 | A03 | Bra012867 | Bn-A04-p1215863 | A04 | Bra014525 |
| Bn-A03-p21980336 | A03 | Bra012838 | Bs_18806_1-p367722 | A04 | Bra003340 |
| Bn-A03-p22679507 | A03 | Bra012698 | Bn-A04-p1472416 | A04 | Bra014564 |
| Bn-A03-p22730208 | A03 | Bra012689 | Bn-A04-p1490485 | A04 | Bra014566 |
| Bn-A03-p23020431 | A03 | Bra012636 | Bn-A04-p1718231 | A04 | Bra014604 |
| Bn-A03-p23562008 | A03 | Bra012558 | Bn-A04-p1869508 | A04 | Bra014629 |
| Bn-A03-p23253636 | A03 | Bra012604 | Bn-A04-p2215376 | A04 | Bra014683 |
| Bn-A03-p23715660 | A03 | Bra012538 | Bn-A04-p2191612 | A04 | Bra014680 |
| Bn-A03-p23185327 | A03 | Bra012617 | Bs_26391_1-p292417 | A04 | Bra014652 |
| Bs_16069_1-p1469430 | A03 | Bra012578 | Bn-A04-p2498254 | A04 | Bra014719 |
| Bn-A03-p24965131 | A03 | Bra019323 | Bn-A04-p3219148 | A04 | Bra007223 |
| Bn-A03-p24016551 | A03 | Bra038794 | Bn-A04-p3262432 | A04 | Bra014802 |
| Bn-A03-p24606883 | A03 | Bra021090 | Bn-A04-p3351067 | A04 | Bra037060 |
| Bn-A03-p24017311 | A03 | Bra038794 | Bn-A04-p3573078 | A04 | Bra014847 |
| Bn-A03-p25265364 | A03 | Bra019284 | Bn-A04-p3571259 | A04 | Bra014847 |
| Bn-A03-p25471848 | A03 | Bra019248 | Bn-A04-p3572717 | A04 | Bra014847 |
| Bn-A03-p25671744 | A03 | Bra019213 | Bn-A04-p4090389 | A04 | Bra033427 |
| Bn-A03-p25668597 | A03 | Bra019213 | Bn-A04-p3631563 | A04 | Bra014856 |
| Bn-A03-p25819955 | A03 | Bra019184 | Bn-A04-p4249132 | A04 | Bra033444 |
| Bn-A03-p25841013 | A03 | Bra019179 | Bn-A04-p3702896 | A04 | Bra039815 |
| Bn-A03-p25879876 | A03 | Bra019171 | Bn-A04-p4529360 | A04 | Bra033482 |
| Bn-A03-p26284449 | A03 | Bra019111 | Bn-A01-p11652789 | A04 | Bra033489 |
| Bn-A03-p26444464 | A03 | Bra019078 | Bn-A04-p6050616 | A04 | Bra033797 |
| Bn-A03-p26437883 | A03 | Bra019079 | Bs_18855_1-p183772 | A04 | Bra033842 |
| Bn-A03-p26800384 | A03 | Bra024225 | Bn-A04-p6008778 | A04 | Bra028133 |
| Bn-A03-p26437565 | A03 | Bra019079 | Bn-A04-p6563107 | A04 | Bra028196 |
| Bn-A03-p27156983 | A03 | Bra024167 | Bs_16394_1-p1502876 | A04 | Bra028195 |
| Bn-A03-p28043216 | A03 | Bra024036 | Bn-A04-p7157859 | A04 | Bra025663 |
| Bn-A04-p7349115 | A04 | Bra025639 | Bn-A04-p18585603 | A04 | Bra039329 |
| Bn-A04-p7350870 | A04 | Bra025639 | Bn-A01-p25175675 | A04 | Bra021452 |
| Bn-A04-p7582986 | A04 | Bra025599 | Bn-A05-p23855991 | A05 | Bra039123 |
| Bn-A04-p7635112 | A04 | Bra025591 | Bn-A05-p23588992 | A05 | Bra040592 |
| Bn-A04-p10602815 | A04 | Bra032145 | Bn-A05-p23607588 | A05 | Bra039179 |
| Bn-A04-p10196289 | A04 | Bra032187 | Bn-A05-p23699894 | A05 | Bra001035 |
| Bn-A04-p8441932 | A04 | Bra025484 | Bn-A02-p26640222 | A05 | Bra032020 |
| Bn-A04-p8178997 | A04 | Bra025518 | Bn-A02-p26832454 | A05 | Bra031979 |
| Bn-A04-p10182453 | A04 | Bra021143 | Bn-A02-p26901243 | A05 | Bra009790 |
| Bn-A04-p9522781 | A04 | Bra013398 | Bs_20376_1-p281604 | A05 | Bra040417 |
| Bn-A04-p9746407 | A04 | Bra030237 | Bs_21496_1-p359451 | A05 | Bra039446 |
| Bn-A04-p10809637 | A04 | Bra032115 | Bn-A05-p23143046 | A05 | Bra029585 |
| Bn-A04-p11274255 | A04 | Bra032044 | Bn-A05-p23030143 | A05 | Bra029606 |
| Bn-A04-p11377702 | A04 | Bra032029 | Bn-A05-p23027881 | A05 | Bra029607 |
| Bn-A04-p11243983 | A04 | Bra032047 | Bn-A05-p22789344 | A05 | Bra029655 |
| Bn-A04-p11164883 | A04 | Bra032063 | Bn-A05-p22973324 | A05 | Bra029615 |
| Bn-A04-p11449464 | A04 | Bra034257 | Bs_17521_1-p837796 | A05 | Bra001245 |
| Bs_24979_1-p134759 | A04 | Bra032035 | Bn-A05-p22599241 | A05 | Bra029700 |
| Bs_25139_1-p441 | A04 | Bra034307 | Bn-A05-p22589874 | A05 | Bra029703 |
| Bs_22278_1-p137755 | A04 | Bra034322 | Bn-A05-p22478538 | A05 | Bra029733 |
| Bn-A04-p11865854 | A04 | Bra034318 | Bn-A05-p22089650 | A05 | Bra029831 |
| Bn-A04-p12677961 | A04 | Bra035705 | Bn-A05-p22111286 | A05 | Bra029829 |
| Bn-A04-p13856134 | A04 | Bra021686 | Bn-A05-p22111345 | A05 | Bra029829 |
| Bn-A04-p13009893 | A04 | Bra035662 | Bn-A05-p21886422 | A05 | Bra029881 |
| Bn-A04-p13389366 | A04 | Bra021607 | Bn-A05-p21463902 | A05 | Bra034798 |
| Bn-A04-p14687930 | A04 | Bra021818 | Bn-A05-p21470614 | A05 | Bra034800 |
| Bn-A04-p14479867 | A04 | Bra021790 | Bn-A05-p21370435 | A05 | Bra034776 |
| Bn-A04-p15485629 | A04 | Bra017301 | Bn-A05-p21370618 | A05 | Bra034776 |
| Bn-A04-p15519877 | A04 | Bra017290 | Bn-A05-p21347821 | A05 | Bra034773 |
| Bn-A04-p15530609 | A04 | Bra017288 | Bn-A05-p20824936 | A05 | Bra027422 |
| Bn-A04-p16268792 | A04 | Bra017159 | Bn-A05-p20503767 | A05 | Bra030830 |
| Bn-A06-p21302070 | A04 | Bra037468 | Bn-A05-p20328443 | A05 | Bra027326 |
| Bn-A04-p16919672 | A04 | Bra017049 | Bn-A05-p20009925 | A05 | Bra027264 |
| Bn-A04-p17170974 | A04 | Bra017008 | Bn-A05-p19622826 | A05 | Bra027195 |
| Bn-A04-p17172246 | A04 | Bra017007 | Bn-A05-p19550645 | A05 | Bra027178 |
| Bn-A04-p17742799 | A04 | Bra016888 | Bs_21015_1-p89425 | A05 | Bra022324 |
| Bn-A04-p17756059 | A04 | Bra016885 | Bn-A05-p19017469 | A05 | Bra022215 |
| Bn-A04-p18156458 | A04 | Bra037697 | Bn-A05-p18453140 | A05 | Bra022316 |
| Bn-A04-p17736387 | A04 | Bra016891 | Bn-A05-p17511337 | A05 | Bra022465 |
| Bn-A04-p17736770 | A04 | Bra016891 | Bs_16263_1-p445748 | A05 | Bra022450 |
| Bn-A04-p17740663 | A04 | Bra016889 | Bn-A05-p16909798 | A05 | Bra036570 |
| Bn-A05-p16898305 | A05 | Bra035745 | Bn-A05-p1134104 | A05 | Bra004630 |
| Bn-A05-p15126127 | A05 | Bra033911 | Bn-A05-p996188 | A05 | Bra004603 |
| Bs_25628_1-p37066 | A05 | Bra033918 | Bn-A05-p917769 | A05 | Bra004588 |
| Bn-A05-p14426042 | A05 | Bra033849 | Bn-A05-p452939 | A05 | Bra004497 |
| Bn-A07-p11390804 | A05 | Bra011912 | Bn-A05-p109998 | A05 | Bra004437 |
| Bn-A05-p9668222 | A05 | Bra037060 | Bn-A05-p114598 | A05 | Bra004438 |
| Bn-A05-p7729367 | A05 | Bra018355 | Bn-A06-p113900 | A06 | Bra038015 |
| Bn-A05-p9623131 | A05 | Bra037055 | Bn-A06-p307436 | A06 | Bra037980 |
| Bn-A05-p7589125 | A05 | Bra018342 | Bn-A06-p362402 | A06 | Bra037970 |
| Bn-A05-p9472646 | A05 | Bra039496 | Bn-A06-p576740 | A06 | Bra039679 |
| Bn-A05-p7062784 | A05 | Bra018272 | Bn-A06-p608570 | A06 | Bra039672 |
| Bn-A05-p7076442 | A05 | Bra018273 | Bn-A06-p621695 | A06 | Bra039669 |
| Bn-A05-p6337412 | A05 | Bra005580 | Bn-A06-p1403969 | A06 | Bra018893 |
| Bn-A05-p6441813 | A05 | Bra005597 | Bn-A06-p1652088 | A06 | Bra018844 |
| Bn-A05-p5521242 | A05 | Bra005445 | Bn-A06-p1636449 | A06 | Bra018846 |
| Bn-A05-p5817231 | A05 | Bra005498 | Bn-A06-p1661477 | A06 | Bra018841 |
| Bn-A05-p5541232 | A05 | Bra005448 | Bn-A06-p2107176 | A06 | Bra018765 |
| Bn-A05-p5714836 | A05 | Bra005475 | Bn-A06-p2234718 | A06 | Bra018743 |
| Bn-A05-p5057096 | A05 | Bra005368 | Bs_16553_1-p34179 | A06 | Bra018731 |
| Bs_15585_1-p978062 | A05 | Bra005350 | Bn-A06-p2437284 | A06 | Bra018706 |
| Bn-A05-p4959282 | A05 | Bra005347 | Bn-A06-p2526552 | A06 | Bra018701 |
| Bs_19248_1-p166341 | A05 | Bra005280 | Bn-A06-p2714610 | A06 | Bra018666 |
| Bn-A05-p4422786 | A05 | Bra005268 | Bn-A06-p2630468 | A06 | Bra018683 |
| Bn-A05-p4316642 | A05 | Bra005255 | Bn-A06-p2717967 | A06 | Bra018665 |
| Bn-A05-p4316943 | A05 | Bra005255 | Bn-A06-p2891175 | A06 | Bra018631 |
| Bn-A05-p3548736 | A05 | Bra005123 | Bn-A06-p2891290 | A06 | Bra018631 |
| Bn-A05-p3467036 | A05 | Bra005108 | Bn-A06-p2778072 | A06 | Bra018657 |
| Bn-A05-p2711007 | A05 | Bra004969 | Bn-A06-p2936438 | A06 | Bra018625 |
| Bn-A05-p2898061 | A05 | Bra004999 | Bn-A06-p3278682 | A06 | Bra020013 |
| Bn-A05-p2653139 | A05 | Bra004957 | Bn-A06-p3066038 | A06 | Bra018593 |
| Bn-A05-p2565586 | A05 | Bra004936 | Bn-A06-p3049884 | A06 | Bra018597 |
| Bn-A05-p2489101 | A05 | Bra004920 | Bn-A06-p3329211 | A06 | Bra019997 |
| Bn-A05-p2494204 | A05 | Bra004920 | Bn-A06-p3496759 | A06 | Bra019960 |
| Bn-A05-p2497466 | A05 | Bra004920 | Bn-A06-p3986320 | A06 | Bra019857 |
| Bn-A05-p1975785 | A05 | Bra004823 | Bn-A06-p4452778 | A06 | Bra019778 |
| Bn-A05-p2353656 | A05 | Bra001421 | Bn-A06-p4419571 | A06 | Bra019782 |
| Bn-A05-p1689277 | A05 | Bra004758 | Bn-A06-p5114238 | A06 | Bra019662 |
| Bn-A05-p1356378 | A05 | Bra004684 | Bn-A06-p5693735 | A06 | Bra026162 |
| Bs_27469_1-p80430 | A05 | Bra004719 | Bn-A06-p6439396 | A06 | Bra026030 |
| Bn-A05-p1372057 | A05 | Bra004691 | Bn-A06-p6679810 | A06 | Bra016603 |
| Bn-A05-p1308471 | A05 | Bra004669 | Bn-A06-p7420466 | A06 | Bra025709 |
| Bn-A06-p7553160 | A06 | Bra025731 | Bn-A06-p21064290 | A06 | Bra037525 |
| Bn-A06-p7927622 | A06 | Bra025778 | Bs_21711_1-p75853 | A06 | Bra025383 |
| Bn-A06-p7931177 | A06 | Bra025778 | Bn-A06-p22088550 | A06 | Bra025342 |
| Bn-A06-p8201495 | A06 | Bra025817 | Bn-A06-p22503987 | A06 | Bra025263 |
| Bn-A06-p8475485 | A06 | Bra012258 | Bn-A06-p22796718 | A06 | Bra025206 |
| Bn-A06-p8472457 | A06 | Bra025857 | Bn-A06-p22808095 | A06 | Bra025202 |
| Bn-A06-p8837251 | A06 | Bra017919 | Bn-A06-p22910339 | A06 | Bra025182 |
| Bn-A06-p9076011 | A06 | Bra017959 | Bn-A06-p23042397 | A06 | Bra025162 |
| Bn-A06-p10046691 | A06 | Bra018079 | Bs_26764_1-p779 | A06 | Bra025162 |
| Bn-A06-p10046575 | A06 | Bra018079 | Bn-A06-p22938934 | A06 | Bra025177 |
| Bn-A06-p10135140 | A06 | Bra018089 | Bn-A06-p23585772 | A06 | Bra024815 |
| Bn-A06-p14316307 | A06 | Bra038638 | Bn-A06-p24167592 | A06 | Bra024897 |
| Bn-A06-p15006796 | A06 | Bra024328 | Bs_15754_1-p730879 | A06 | Bra024900 |
| Bn-A06-p15262271 | A06 | Bra024368 | Bn-A06-p24319329 | A06 | Bra024940 |
| Bn-A06-p15375170 | A06 | Bra024381 | Bs_19724_1-p749258 | A06 | Bra025028 |
| Bn-A06-p15421020 | A06 | Bra024386 | Bn-A06-p25351980 | A06 | Bra033732 |
| Bn-A06-p15420581 | A06 | Bra024386 | Bn-A06-p26152237 | A06 | Bra033575 |
| Bn-A06-p15893014 | A06 | Bra024456 | Bn-A07-p22398500 | A07 | Bra003583 |
| Bn-A06-p15893221 | A06 | Bra024456 | Bn-A07-p22362597 | A07 | Bra008434 |
| Bn-A06-p16406113 | A06 | Bra009654 | Bn-A07-p22250567 | A07 | Bra035119 |
| Bn-A06-p16461885 | A06 | Bra009669 | Bn-A07-p22050904 | A07 | Bra035077 |
| Bn-A06-p17037912 | A06 | Bra009754 | Bn-A07-p21569289 | A07 | Bra015646 |
| Bn-A06-p17007775 | A06 | Bra009750 | Bn-A07-p21577164 | A07 | Bra015645 |
| Bn-A06-p17337842 | A06 | Bra009798 | Bn-A07-p22005058 | A07 | Bra035065 |
| Bn-A06-p17175388 | A06 | Bra009774 | Bn-A07-p20999615 | A07 | Bra015758 |
| Bn-A06-p17549603 | A06 | Bra009827 | Bn-A07-p20230189 | A07 | Bra015917 |
| Bn-A06-p17684443 | A06 | Bra009854 | Bn-A07-p19977445 | A07 | Bra003835 |
| Bn-A06-p17741868 | A06 | Bra009861 | Bn-A07-p19915062 | A07 | Bra015993 |
| Bn-A06-p17778210 | A06 | Bra009866 | Bn-A07-p16923977 | A07 | Bra002062 |
| Bn-A06-p17778079 | A06 | Bra009866 | Bn-A07-p16925201 | A07 | Bra004116 |
| Bn-A06-p18500625 | A06 | Bra009992 | Bn-A07-p16970000 | A07 | Bra004123 |
| Bs_17291_1-p732305 | A06 | Bra010036 | Bn-A07-p16146639 | A07 | Bra003989 |
| Bn-A06-p18706102 | A06 | Bra010026 | Bn-A07-p15885711 | A07 | Bra003943 |
| Bn-A06-p21268967 | A06 | Bra037477 | Bn-A07-p15829285 | A07 | Bra003934 |
| Bn-A06-p21162770 | A06 | Bra037501 | Bn-A07-p14958606 | A07 | Bra003762 |
| Bn-A06-p21252883 | A06 | Bra037481 | Bn-A07-p14792267 | A07 | Bra003738 |
| Bn-A06-p21164284 | A06 | Bra037501 | Bn-A07-p14193108 | A07 | Bra003640 |
| Bn-A06-p21101957 | A06 | Bra037517 | Bn-A07-p13926029 | A07 | Bra003584 |
| Bn-A06-p21064129 | A06 | Bra037525 | Bn-A07-p13828973 | A07 | Bra003567 |
| Bn-A06-p21065019 | A06 | Bra037525 | Bn-A07-p13715621 | A07 | Bra003544 |
| Bn-A06-p21064341 | A06 | Bra037525 | Bn-A07-p13712520 | A07 | Bra003543 |
| Bn-A07-p13712881 | A07 | Bra003543 | Bn-A07-p6542254 | A07 | Bra032912 |
| Bn-A07-p13485413 | A07 | Bra003496 | Bn-A07-p6494007 | A07 | Bra030111 |
| Bn-A07-p13639374 | A07 | Bra003527 | Bn-A07-p6373884 | A07 | Bra010847 |
| Bn-A07-p13654191 | A07 | Bra003533 | Bn-A07-p6262438 | A07 | Bra030137 |
| Bn-A07-p13512124 | A07 | Bra003505 | Bs_17326_1-p718434 | A07 | Bra030137 |
| Bn-A07-p12734895 | A07 | Bra003347 | Bn-A07-p5670936 | A07 | Bra032378 |
| Bn-A07-p12751218 | A07 | Bra003349 | Bs_17580_1-p160243 | A07 | Bra014879 |
| Bn-A07-p12878986 | A07 | Bra003368 | Bn-A01-p10518110 | A07 | Bra034933 |
| Bs_15763_1-p262418 | A07 | Bra003366 | Bn-A01-p10513529 | A07 | Bra026229 |
| Bn-A07-p11572985 | A07 | Bra003146 | Bn-A07-p3202245 | A07 | Bra015135 |
| Bn-A02-p771313 | A07 | Bra028506 | Bn-A07-p4817318 | A07 | Bra014964 |
| Bn-A02-p474724 | A07 | Bra028460 | Bn-A07-p4155337 | A07 | Bra015072 |
| Bn-A02-p126006 | A07 | Bra028409 | Bn-A07-p4815910 | A07 | Bra014964 |
| Bn-A07-p10891272 | A07 | Bra011965 | Bn-A10-p12072657 | A07 | Bra002028 |
| Bs_16402_1-p76139 | A07 | Bra011923 | Bn-A10-p12014652 | A07 | Bra014231 |
| Bn-A07-p10848190 | A07 | Bra011972 | Bn-A10-p11918400 | A07 | Bra022042 |
| Bn-A07-p11333494 | A07 | Bra007227 | Bn-A10-p11914509 | A07 | Bra025290 |
| Bn-A07-p11044717 | A07 | Bra011948 | Bn-A10-p11637847 | A07 | Bra002080 |
| Bn-A07-p10750190 | A07 | Bra011986 | Bn-A07-p1725633 | A07 | Bra039908 |
| Bn-A07-p10755129 | A07 | Bra011986 | Bn-A07-p1217535 | A07 | Bra038830 |
| Bn-A07-p10673477 | A07 | Bra011997 | Bs_25323_1-p7608 | A07 | Bra014149 |
| Bs_18505_1-p254578 | A07 | Bra011997 | Bn-A07-p1255437 | A07 | Bra038827 |
| Bn-A07-p10418835 | A07 | Bra012043 | Bn-A07-p831443 | A07 | Bra006858 |
| Bn-A07-p10347794 | A07 | Bra011913 | Bn-A07-p244051 | A07 | Bra036463 |
| Bn-A07-p10209154 | A07 | Bra012069 | Bn-A07-p132150 | A07 | Bra035662 |
| Bn-A07-p10099786 | A07 | Bra012087 | Bn-A08-p211090 | A08 | Bra030845 |
| Bn-A07-p9841369 | A07 | Bra012134 | Bn-A08-p758590 | A08 | Bra030915 |
| Bn-A07-p9034194 | A07 | Bra012258 | Bn-A08-p940619 | A08 | Bra030935 |
| Bn-A07-p9523515 | A07 | Bra012180 | Bn-A08-p798970 | A08 | Bra030921 |
| Bn-A07-p9079474 | A07 | Bra012251 | Bn-A08-p1847328 | A08 | Bra014298 |
| Bn-A07-p8959937 | A07 | Bra016434 | Bn-A08-p1088701 | A08 | Bra030958 |
| Bn-A07-p8259212 | A07 | Bra012357 | Bs_16755_1-p1427195 | A08 | Bra030946 |
| Bn-A07-p8258521 | A07 | Bra012357 | Bn-A08-p2275410 | A08 | Bra014240 |
| Bn-A07-p8324017 | A07 | Bra012343 | Bn-A08-p2425488 | A08 | Bra014221 |
| Bn-A07-p8560948 | A07 | Bra031383 | Bs_17807_1-p461276 | A08 | Bra014136 |
| Bn-A07-p8790021 | A07 | Bra012289 | Bs_16287_1-p395887 | A08 | Bra038423 |
| Bn-A07-p8037081 | A07 | Bra016328 | Bn-A08-p4528943 | A08 | Bra014018 |
| Bn-A07-p6906296 | A07 | Bra010924 | Bn-A08-p4837890 | A08 | Bra013993 |
| Bn-A07-p6942882 | A07 | Bra030054 | Bn-A08-p4254041 | A08 | Bra014039 |
| Bn-A07-p7124154 | A07 | Bra012506 | Bn-A06-p17681092 | A08 | Bra018293 |
| Bn-A07-p7027400 | A07 | Bra030034 | Bn-A08-p8426380 | A08 | Bra038944 |
| Bn-A08-p6488809 | A08 | Bra039712 | Bn-A08-p20244218 | A08 | Bra016839 |
| Bn-A08-p6268593 | A08 | Bra039856 | Bn-A08-p20441889 | A08 | Bra030765 |
| Bn-A08-p6170057 | A08 | Bra030091 | Bn-A05-p8290002 | A08 | Bra018454 |
| Bn-A08-p6828854 | A08 | Bra038063 | Bn-A08-p20717142 | A08 | Bra030706 |
| Bn-A08-p6828857 | A08 | Bra038063 | Bn-A08-p20839304 | A08 | Bra030679 |
| Bn-A08-p6829643 | A08 | Bra038063 | Bn-A08-p21330967 | A08 | Bra030558 |
| Bn-A08-p10067927 | A08 | Bra026230 | Bn-A08-p21228678 | A08 | Bra030581 |
| Bn-A08-p10123561 | A08 | Bra007196 | Bn-A08-p21227076 | A08 | Bra030581 |
| Bn-A08-p11308315 | A08 | Bra020904 | Bn-A09-p36891502 | A09 | Bra039495 |
| Bn-A08-p11352069 | A08 | Bra020895 | Bn-A09-p36619537 | A09 | Bra033370 |
| Bs_17119_1-p85292 | A08 | Bra040065 | Bn-A09-p36136583 | A09 | Bra032450 |
| Bn-A08-p12761022 | A08 | Bra034574 | Bn-A09-p35753486 | A09 | Bra031576 |
| Bn-A08-p13239816 | A08 | Bra010217 | Bs_16389_1-p215602 | A09 | Bra031538 |
| Bn-A08-p14156050 | A08 | Bra010368 | Bn-A09-p35510262 | A09 | Bra031627 |
| Bn-A08-p14749890 | A08 | Bra013843 | Bn-A09-p35485049 | A09 | Bra031632 |
| Bn-A08-p14447288 | A08 | Bra010420 | Bn-A09-p35485458 | A09 | Bra031632 |
| Bn-A08-p14496135 | A08 | Bra010432 | Bn-A09-p35672030 | A09 | Bra031592 |
| Bn-A08-p14500962 | A08 | Bra010432 | Bn-A09-p35466517 | A09 | Bra031637 |
| Bn-A08-p15326882 | A08 | Bra010584 | Bn-A09-p34995048 | A09 | Bra031737 |
| Bn-A08-p15273100 | A08 | Bra010574 | Bn-A09-p34482075 | A09 | Bra026957 |
| Bn-A08-p16028375 | A08 | Bra010694 | Bn-A09-p34067269 | A09 | Bra026871 |
| Bn-A08-p16317873 | A08 | Bra010767 | Bs_16445_1-p2013455 | A09 | Bra026749 |
| Bn-A08-p16344396 | A08 | Bra010773 | Bs_16445_1-p1834116 | A09 | Bra026780 |
| Bn-A08-p16771030 | A08 | Bra010847 | Bs_16445_1-p1839966 | A09 | Bra026779 |
| Bn-A08-p17381323 | A08 | Bra010978 | Bn-A09-p33499505 | A09 | Bra026105 |
| Bn-A08-p17291598 | A08 | Bra010960 | Bn-A09-p33428597 | A09 | Bra026728 |
| Bn-A08-p17217296 | A08 | Bra010940 | Bn-A09-p33427256 | A09 | Bra026728 |
| Bn-A08-p17256848 | A08 | Bra010953 | Bs_16445_1-p2549752 | A09 | Bra030991 |
| Bn-A08-p18353208 | A08 | Bra016416 | Bn-A09-p32937856 | A09 | Bra025932 |
| Bn-A08-p17472208 | A08 | Bra010992 | Bn-A09-p32275168 | A09 | Bra031153 |
| Bn-A08-p17586676 | A08 | Bra011014 | Bs_16197_1-p225455 | A09 | Bra006858 |
| Bn-A08-p17639419 | A08 | Bra016271 | Bn-A09-p31979689 | A09 | Bra031215 |
| Bn-A08-p18630178 | A08 | Bra016471 | Bn-A09-p31982487 | A09 | Bra031214 |
| Bn-A08-p19173201 | A08 | Bra016588 | Bn-A09-p30780679 | A09 | Bra007763 |
| Bn-A08-p19284258 | A08 | Bra016611 | Bn-A09-p30877241 | A09 | Bra007787 |
| Bn-A08-p19346186 | A08 | Bra016630 | Bn-A09-p30711004 | A09 | Bra007749 |
| Bn-A08-p19454590 | A08 | Bra016655 | Bn-A09-p5062101 | A09 | Bra038521 |
| Bn-A08-p19504101 | A08 | Bra016669 | Bn-A09-p30654305 | A09 | Bra023649 |
| Bn-A08-p19578180 | A08 | Bra026200 | Bn-A09-p30284632 | A09 | Bra007651 |
| Bn-A08-p19626269 | A08 | Bra016707 | Bn-A09-p30371768 | A09 | Bra007680 |
| Bn-A08-p20085504 | A08 | Bra016811 | Bn-A09-p30437673 | A09 | Bra007695 |
| Bn-A09-p30129045 | A09 | Bra007628 | Bn-A09-p13667337 | A09 | Bra027515 |
| Bn-A09-p30032125 | A09 | Bra007611 | Bn-A09-p13693499 | A09 | Bra027512 |
| Bn-A09-p30105467 | A09 | Bra007626 | Bs_18100_1-p593993 | A09 | Bra027485 |
| Bn-A09-p29629155 | A09 | Bra007527 | Bn-A09-p9789898 | A09 | Bra027913 |
| Bn-A09-p29508036 | A09 | Bra007497 | Bn-A09-p10092753 | A09 | Bra027955 |
| Bn-A09-p28723813 | A09 | Bra007345 | Bs_16912_1-p28213 | A09 | Bra027897 |
| Bn-A09-p28212233 | A09 | Bra007252 | Bn-A09-p9371106 | A09 | Bra027866 |
| Bs_16361_1-p952834 | A09 | Bra007253 | Bn-A09-p8635777 | A09 | Bra027121 |
| Bn-A09-p28060237 | A09 | Bra007221 | Bn-A09-p7560188 | A09 | Bra027009 |
| Bn-A09-p28149545 | A09 | Bra007238 | Bn-A09-p6632506 | A09 | Bra027723 |
| Bn-A04-p2309625 | A09 | Bra014696 | Bn-A09-p6616405 | A09 | Bra027726 |
| Bn-A09-p28209274 | A09 | Bra007251 | Bn-A09-p7490528 | A09 | Bra027004 |
| Bn-A09-p28026997 | A09 | Bra007213 | Bn-A09-p7318713 | A09 | Bra027621 |
| Bn-A09-p27351028 | A09 | Bra007105 | Bn-A09-p5678953 | A09 | Bra036682 |
| Bn-A07-p6929108 | A09 | Bra032698 | Bs_17487_1-p723169 | A09 | Bra036682 |
| Bn-A09-p27869414 | A09 | Bra007189 | Bn-A09-p5431352 | A09 | Bra036721 |
| Bn-A09-p26577478 | A09 | Bra006968 | Bn-A09-p4433127 | A09 | Bra037304 |
| Bn-A09-p26142571 | A09 | Bra006917 | Bs_17487_1-p160800 | A09 | Bra038835 |
| Bn-A09-p26054532 | A09 | Bra006900 | Bn-A09-p4835780 | A09 | Bra037238 |
| Bn-A09-p23325795 | A09 | Bra024724 | Bn-A09-p4288843 | A09 | Bra037142 |
| Bs_16770_1-p3050704 | A09 | Bra036818 | Bn-A09-p4164576 | A09 | Bra037169 |
| Bn-A09-p23436500 | A09 | Bra024699 | Bn-A09-p4416731 | A09 | Bra037112 |
| Bn-A09-p25779397 | A09 | Bra036791 | Bs_17423_1-p100318 | A09 | Bra031821 |
| Bn-A09-p25618420 | A09 | Bra036818 | Bs_17190_1-p237474 | A09 | Bra035837 |
| Bn-A09-p25004061 | A09 | Bra036053 | Bn-A01-p9080359 | A09 | Bra026476 |
| Bs_16181_1-p45725 | A09 | Bra032344 | Bn-A09-p1558529 | A09 | Bra036521 |
| Bn-A09-p22462771 | A09 | Bra032333 | Bn-A09-p2044817 | A09 | Bra036620 |
| Bn-A09-p21865966 | A09 | Bra032385 | Bn-A09-p1243032 | A09 | Bra039077 |
| Bn-A09-p20720084 | A09 | Bra023204 | Bn-A01-p26760158 | A09 | Bra034246 |
| Bn-A09-p19545956 | A09 | Bra027997 | Bn-A01-p26921219 | A09 | Bra034208 |
| Bs_17740_1-p411057 | A09 | Bra037853 | Bn-A01-p27017324 | A09 | Bra034192 |
| Bn-A01-p8782649 | A09 | Bra039768 | Bn-A09-p301830 | A09 | Bra036283 |
| Bn-A09-p17723940 | A09 | Bra029475 | Bs_22749_1-p202280 | A09 | Bra036283 |
| Bn-A07-p5757189 | A09 | Bra014868 | Bs_15783_1-p501489 | A09 | Bra036283 |
| Bs_18424_1-p672600 | A09 | Bra017345 | Bn-A09-p302062 | A09 | Bra036283 |
| Bn-A09-p15005673 | A09 | Bra017400 | Bn-A09-p219538 | A09 | Bra036271 |
| Bn-A09-p15270033 | A09 | Bra008515 | Bn-A09-p788595 | A09 | Bra037381 |
| Bs_23293_1-p239190 | A09 | Bra017429 | Bn-A09-p869372 | A09 | Bra037364 |
| Bn-A09-p15960945 | A09 | Bra032938 | Bn-A10-p16824950 | A10 | Bra009470 |
| Bn-A09-p16835122 | A09 | Bra017600 | Bs_21636_1-p137494 | A10 | Bra009483 |
| Bn-A09-p13253434 | A09 | Bra027574 | Bs_21636_1-p137408 | A10 | Bra009483 |
| Bn-A10-p16712853 | A10 | Bra009453 | Bn-A10-p13162021 | A10 | Bra008630 |
| Bn-A10-p16674476 | A10 | Bra009444 | Bn-A10-p13130002 | A10 | Bra008624 |
| Bn-A10-p15237975 | A10 | Bra009096 | Bn-A10-p13008713 | A10 | Bra008602 |
| Bn-A10-p15433413 | A10 | Bra009150 | Bn-A10-p12999930 | A10 | Bra008600 |
| Bn-A10-p15408188 | A10 | Bra009143 | Bn-A10-p13019909 | A10 | Bra006366 |
| Bn-A10-p15401999 | A10 | Bra009141 | Bn-A10-p12910850 | A10 | Bra008580 |
| Bn-A10-p15327377 | A10 | Bra009123 | Bn-A10-p10680827 | A10 | Bra036737 |
| Bn-A10-p15542820 | A10 | Bra009177 | Bn-A10-p10651872 | A10 | Bra002253 |
| Bn-A10-p15607468 | A10 | Bra009196 | Bn-A10-p10982591 | A10 | Bra002191 |
| Bn-A10-p15729205 | A10 | Bra009227 | Bn-A10-p10186330 | A10 | Bra035167 |
| Bn-A10-p15719803 | A10 | Bra009223 | Bn-A10-p9436205 | A10 | Bra002458 |
| Bn-A10-p15742287 | A10 | Bra009230 | Bn-A10-p9630194 | A10 | Bra002427 |
| Bn-A10-p15606935 | A10 | Bra009196 | Bn-A10-p9186877 | A10 | Bra002503 |
| Bs_17750_1-p823170 | A10 | Bra009196 | Bn-A10-p9181902 | A10 | Bra002504 |
| Bn-A10-p15924608 | A10 | Bra009274 | Bn-A10-p8951109 | A10 | Bra020303 |
| Bn-A08-p14644041 | A10 | Bra010462 | Bn-A10-p8984096 | A10 | Bra002538 |
| Bn-A10-p16109898 | A10 | Bra009310 | Bn-A10-p8503752 | A10 | Bra002625 |
| Bn-A10-p16312405 | A10 | Bra009354 | Bn-A10-p8766754 | A10 | Bra037954 |
| Bn-A10-p15160966 | A10 | Bra009079 | Bn-A10-p8767803 | A10 | Bra002581 |
| Bn-A10-p16344917 | A10 | Bra009363 | Bn-A10-p8340529 | A10 | Bra002664 |
| Bs_17109_2-p188015 | A10 | Bra009028 | Bn-A10-p8285049 | A10 | Bra002679 |
| Bn-A10-p15021776 | A10 | Bra009047 | Bn-A10-p8408212 | A10 | Bra002645 |
| Bn-A10-p15031810 | A10 | Bra009051 | Bn-A10-p7906281 | A10 | Bra002744 |
| Bn-A10-p14883536 | A10 | Bra009018 | Bn-A10-p7767853 | A10 | Bra002762 |
| Bn-A10-p14394392 | A10 | Bra008916 | Bn-A10-p7905059 | A10 | Bra002744 |
| Bn-A10-p14373433 | A10 | Bra008908 | Bn-A10-p7341107 | A10 | Bra002825 |
| Bn-A10-p14694580 | A10 | Bra008974 | Bn-A10-p7326339 | A10 | Bra002826 |
| Bn-A10-p14658742 | A10 | Bra008966 | Bn-A02-p22478377 | A10 | Bra036329 |
| Bn-A10-p14687193 | A10 | Bra008972 | Bn-A10-p515442 | A10 | Bra015620 |
| Bn-A10-p14693723 | A10 | Bra008973 | Bs_18140_1-p536327 | A10 | Bra015597 |
| Bn-A10-p14334390 | A10 | Bra008895 | Bn-A10-p1125100 | A10 | Bra015549 |
| Bn-A10-p14092948 | A10 | Bra008846 | Bn-A10-p1125156 | A10 | Bra015549 |
| Bn-A10-p14012013 | A10 | Bra008827 | Bn-A10-p1264986 | A10 | Bra015522 |
| Bn-A10-p13997359 | A10 | Bra008825 | Bn-A10-p1273491 | A10 | Bra015521 |
| Bn-A10-p13932541 | A10 | Bra008809 | Bn-A10-p1215054 | A10 | Bra015529 |
| Bn-A10-p13875041 | A10 | Bra008798 | Bn-A10-p1215039 | A10 | Bra015529 |
| Bn-A10-p13659996 | A10 | Bra008742 | Bn-A10-p2309584 | A10 | Bra015355 |
| Bn-A10-p13471114 | A10 | Bra008703 | Bs_16414_1-p863783 | A10 | Bra015291 |
| Bs_22082_1-p906323 | A10 | Bra008712 | Bs_16414_1-p1031719 | A10 | Bra033410 |
| Bs_16362_1-p91070 | A10 | Bra008702 | Bn-A10-p3845546 | A10 | Bra033404 |
| Bn-A10-p13577876 | A10 | Bra008723 | Bn-A10-p2805505 | A10 | Bra015264 |

Table S3. Details of the 135 InDel/SNP markers on the map of F2-485

| **Marker** | **Type** | **Position** | **Physical distance** | **Marker** | **Length** | **Common marker** |
| --- | --- | --- | --- | --- | --- | --- |
| B01-15-2 | SNP | A01 | 994572 | - | 149 | Bn-A01-p994722-1 |
| zyj1-1-2 | InDel | A01 | 1214145 | - | 158 |  |
| zyj1-2-2 | InDel | A01 | 1719430 | - | 95 |  |
| zyj1-4-2 | InDel | A01 | 3416399 | - | 153 |  |
| A01-13-1 | InDel | A01 | 5717292 | BrID10985 | 122 |  |
| ZYJ1-7-2 | InDel | A01 | 6295028 | - | 151 |  |
| B01-10-1 | SNP | A01 | 6385820 | - | 130 | Bn-A01-p6376105-2 |
| ZYJ1-8 | InDel | A01 | 7148991 | - | 92 |  |
| ZYJ1-9 | InDel | A01 | 7759260 | - | 129 |  |
| A01-15 | InDel | A01 | 8554140 | BrID10307 | 100 |  |
| B01-8-1 | SNP | A01 | 9011487 | - | 152 | Bn-A01-p9223420-1 |
| zyj1-12-2 | InDel | A01 | 10873391 | - | 157 |  |
| ZYJ1-11-2 | InDel | A01 | 13997329 | - | 132 |  |
| ZYJ1-15-2 | InDel | A01 | 16097941 | - | 149 |  |
| B01-2-1 | SNP | A01 | 16379832 | - | 177 | Bn-A01-p16537866-1 |
| zyj1-16-2 | InDel | A01 | 18280370 | - | 122 |  |
| A01-7 | InDel | A01 | 21426514 | BrID101201 | 140 |  |
| B01-5-2 | SNP | A01 | 23828365 | - | 130 | Bn-A01-p24655110-1 |
| A01-8 | InDel | A01 | 23841158 | BrID10433 | 89 |  |
| A01-29 | InDel | A01 | 27992199 | BrID10879 | 84 |  |
| B02-2-1 | SNP | A02 | 2389248 | - | 101 | Bn-A02-p3422005-1 |
| A02-19-1 | InDel | A02 | 14949619 | BrID10557 | 100 |  |
| A02-6-2 | InDel | A02 | 18786636 | BrID10447 | 81 |  |
| B02-5-1 | SNP | A02 | 19351081 | - | 118 | Bn-A02-p19683093-1 |
| zyj2-9-1 | InDel | A02 | 21774955 | - | 103 |  |
| zyj2-10-2 | InDel | A02 | 22431954 | - | 130 |  |
| ZYJ2-12-2 | InDel | A02 | 23906823 | - | 117 |  |
| B02-7-1 | SNP | A02 | 24633733 | - | 111 | Bn-A02-p25181474-1 |
| A02-10-2 | InDel | A02 | 27009145 | BrID101169 | 134 |  |
| zyj3-3-2 | InDel | A03 | 3563205 | - | 96 |  |
| B03-11-1 | SNP | A03 | 5444150 | - | 156 | Bn-A03-p5439400-1 |
| B03-12-2 | SNP | A03 | 6929149 | - | 128 | Bn-A03-p6919500-3 |
| B03-3-2 | SNP | A03 | 8639644 | - | 123 | Bn-A03-p8629992-1 |
| A03-6 | InDel | A03 | 15083012 | BrID90424 | 200 |  |
| B03-9-2 | SNP | A03 | 16832453 | - | 101 | Bn-A03-p16812953-1 |
| zyj3-13-2 | InDel | A03 | 19486527 | - | 100 |  |
| zyj3-14-2 | InDel | A03 | 20422869 | - | 109 |  |
| B03-15-2 | SNP | A03 | 20584919 | - | 149 | Bn-A03-p20560569-2 |
| A03-29 | InDel | A03 | 20786650 | BrID10289 | 98 |  |
| A03-13-2 | InDel | A03 | 29633644 | BrID10367 | 97 |  |
| A03-10-2 | InDel | A03 | 30002046 | BrID90131 | 191 |  |
| A04-13-2 | InDel | A04 | 2417875 | BrID10321 | 84 |  |
| A04-17-2 | InDel | A04 | 7441204 | BrID10645 | 87 |  |
| ZYJ4-7 | InDel | A04 | 9481614 | - | 108 |  |
| A04-6 | InDel | A04 | 9603024 | BrID90143 | 191 |  |
| ZYJ4-9-2 | InDel | A04 | 13560249 | - | 105 |  |
| zyj5-1-2 | InDel | A05 | 1631177 | - | 131 |  |
| B05-6-1 | SNP | A05 | 1671671 | - | 109 | Bn-A05-p1671823-1 |
| A05-41-2 | InDel | A05 | 2611249 | BrID10511 | 100 |  |
| B05-7-1 | SNP | A05 | 2897911 | - | 114 | Bn-A05-p2898061-1 |
| zyj5-5 | InDel | A05 | 3977221 | - | 122 |  |
| B05-15-2 | SNP | A05 | 6337262 | - | 116 | Bn-A05-p6337412-1 |
| zyj5-6 | InDel | A05 | 6340979 | - | 143 |  |
| zyj5-9 | InDel | A05 | 10519045 | - | 152 |  |
| zyj5-15-2 | InDel | A05 | 17339857 | - | 120 |  |
| zyj5-16-2 | InDel | A05 | 18659064 | - | 126 |  |
| A05-7 | InDel | A05 | 19069709 | BrID10583 | 91 |  |
| A05-22-1 | InDel | A05 | 19168911 | BrID10581 | 99 |  |
| B05-2-1 | SNP | A05 | 19169597 |  | 129 | Bn-A05-p18453140-1 |
| zyj5-17-1 | InDel | A05 | 19188294 | - | 151 |  |
| zyj5-18 | InDel | A05 | 19924523 | - | 116 |  |
| A05-16-2 | InDel | A05 | 21640807 | BrID101183 | 93 |  |
| ZYJ5-20-2 | InDel | A05 | 21807146 | - | 96 |  |
| A06-1-2 | InDel | A06 | 124076 | BrID90105 | 179 |  |
| A06-12 | InDel | A06 | 607068 | BrID10865 | 99 |  |
| A06-14-2 | InDel | A06 | 952461 | BrID101049 | 140 |  |
| A06-15-1 | InDel | A06 | 1275788 | BrID10997 | 128 |  |
| ZYJ6-4-2 | InDel | A06 | 3065281 | - | 152 |  |
| B06-2-2 | SNP | A06 | 4467328 | - | 133 | Bn-A06-p4452778-1 |
| A06-4 | InDel | A06 | 5117471 | BrID10407 | 95 |  |
| ZYJ6-6-2 | InDel | A06 | 6332925 | - | 132 |  |
| A06-22-2 | InDel | A06 | 6360355 | BrID101163 | 100 |  |
| A06-25-2 | InDel | A06 | 7664998 | BrID10649 | 80 |  |
| A06-40-2 | InDel | A06 | 8802051 | BrID90005 | 126 |  |
| A06-42 | InDel | A06 | 10207312 | BrID10375 | 90 |  |
| A06-26-2 | InDel | A06 | 12533827 | BrID90400 | 200 |  |
| A06-30-1 | InDel | A06 | 19519825 | BrID101089 | 139 |  |
| B06-4-1 | SNP | A06 | 21837515 | - | 111 | Bn-A06-p22910339-1 |
| zyj6-19-2 | InDel | A06 | 22510965 | - | 149 |  |
| A07-11 | InDel | A07 | 141310 | BrID10811 | 98 |  |
| zyj7-1-2 | InDel | A07 | 166456 | - | 140 |  |
| A07-15 | InDel | A07 | 3872313 | BrID10329 | 84 |  |
| A07-14 | InDel | A07 | 4047366 | BrID10327 | 81 |  |
| A07-2 | InDel | A07 | 4496925 | BrID90199 | 191 |  |
| zyj7-6-1 | InDel | A07 | 7230852 | - | 160 |  |
| zyj7-7-1 | InDel | A07 | 8483221 | - | 151 |  |
| A07-3-2 | InDel | A07 | 8890847 | BrID90043 | 187 |  |
| B07-12-2 | SNP | A07 | 9249510 | - | 149 | Bn-A07-p7027400-1 |
| A07-21-1 | InDel | A07 | 11667757 | BrID10487 | 93 |  |
| B07-3-1 | SNP | A07 | 12326797 | - | 159 | Bn-A07-p10099786-1 |
| zyj7-9-2 | InDel | A07 | 12815346 | - | 155 |  |
| A07-5-2 | InDel | A07 | 13590597 | BrID10497 | 100 |  |
| zyj7-11 | InDel | A07 | 14793452 | - | 158 |  |
| zyj7-12-1 | InDel | A07 | 15862437 | - | 137 |  |
| B07-6-1 | SNP | A07 | 16917746 | - | 140 | Bn-A07-p13639374-1 |
| B07-8-2 | SNP | A07 | 18236928 | - | 116 | Bn-A07-p14958606-1 |
| A07-40 | InDel | A07 | 19873576 | BrID10347 | 94 |  |
| zyj7-16-1 | InDel | A07 | 20894341 | - | 136 |  |
| B07-9-2 | SNP | A07 | 23260717 | - | 120 | Bn-A07-p19977445-1 |
| zyj7-20 | InDel | A07 | 24983174 | - | 155 |  |
| A08-1-1 | InDel | A08 | 430450 | BrID10727 | 97 |  |
| zyj8-2-2 | InDel | A08 | 4207813 | - | 146 |  |
| B08-3-2 | SNP | A08 | 4533693 | - | 138 | Bn-A08-p4528943-1 |
| A08-5-2 | InDel | A08 | 11839098 | BrID10933 | 140 |  |
| B08-11-2 | SNP | A08 | 11971667 | - | 131 | Bn-A08-p12761022-1 |
| B08-7-1 | SNP | A08 | 13667733 | - | 182 | Bn-A08-p14447288-1 |
| zyj8-9-2 | InDel | A08 | 14017919 | - | 86 |  |
| B08-1-2 | SNP | A08 | 16807121 | - | 153 | Bn-A08-p17586676-1 |
| B08-2-1 | SNP | A08 | 19310849 | - | 124 | Bn-A08-p20085504-1 |
| A09-2-1 | InDel | A09 | 1211102 | BrID10853 | 92 |  |
| B09-17-2 | SNP | A09 | 1594248 | - | 123 | Bn-A09-p1243032-5 |
| B09-7-1 | SNP | A09 | 2537238 | - | 111 | Bn-A09-p2044817-1 |
| ZYJ9-5-1 | InDel | A09 | 5137862 | - | 144 |  |
| A09-4-1 | InDel | A09 | 6684130 | BrID90319 | 194 |  |
| B09-15-2 | SNP | A09 | 7056965 | - | 117 | Bn-A09-p6632506-1 |
| ZYJ9-7-2 | InDel | A09 | 12459199 | - | 121 |  |
| A09-37-2 | InDel | A09 | 20045431 | BrID10613 | 87 |  |
| ZYJ9-9-2 | InDel | A09 | 21909939 | - | 136 |  |
| ZYJ9-10-2 | InDel | A09 | 22501505 | - | 155 |  |
| A09-29-1 | InDel | A09 | 24846233 | BrID10955 | 138 |  |
| A09-24-1 | InDel | A09 | 27043201 | BrID10541 | 92 |  |
| A09-8-2 | InDel | A09 | 29276890 | BrID10547 | 93 |  |
| B09-8-1 | SNP | A09 | 30862771 | - | 113 | Bn-A09-p29348596-1 |
| ZYJ9-17-2 | InDel | A09 | 30894030 | - | 155 |  |
| A09-9-1 | InDel | A09 | 31323402 | BrID101217 | 100 |  |
| B09-13-1 | SNP | A09 | 31516922 | - | 157 | Bn-A09-p30002747-1 |
| A09-18-2 | InDel | A09 | 32066148 | BrID90071 | 163 |  |
| A10-1-2 | InDel | A10 | 190810 | BrID10833 | 99 |  |
| A10-37-2 | InDel | A10 | 372402 | BrID101023 | 117 |  |
| zyj10-4-2 | InDel | A10 | 3775136 | - | 145 |  |
| zyj10-5-2 | InDel | A10 | 5027036 | - | 105 |  |
| B10-4-2 | SNP | A10 | 9251589 | - | 128 | Bn-A10-p9186877-1 |
| B10-6-1 | SNP | A10 | 13835913 | - | 100 | Bn-A10-p15031810-1 |
| A10-7 | InDel | A10 | 14146327 | BrID10567 | 81 |  |
| A10-9 | InDel | A10 | 16385224 | BrID10577 | 92 |  |


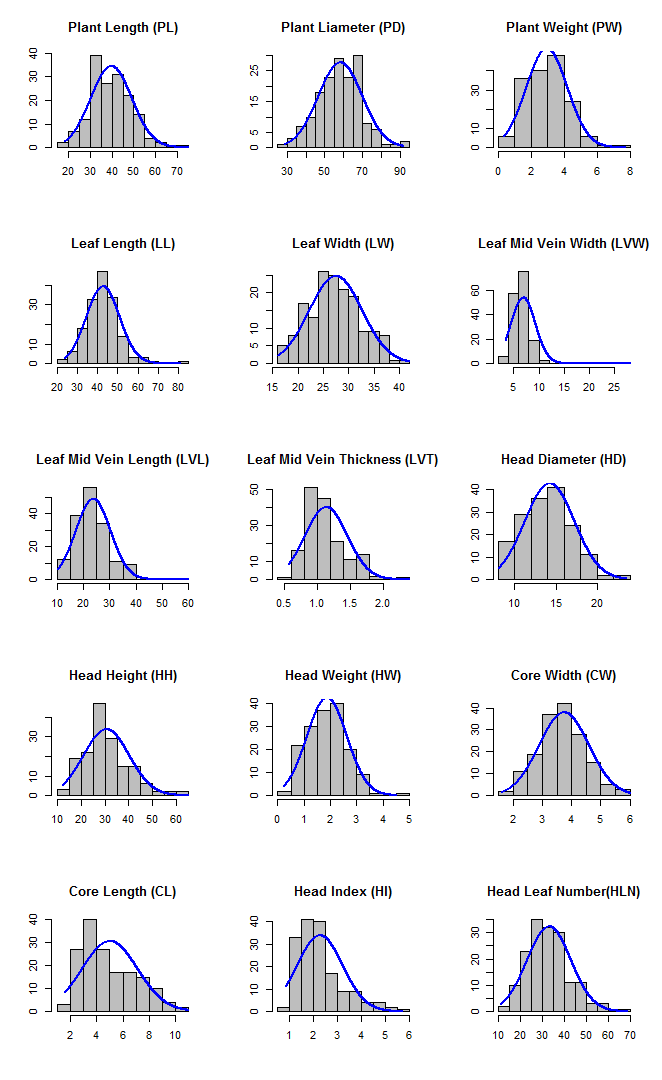
Figure S1. Distribution analysis for PL, PD, PW, LL, LW, LVW, LVL, LVT, HD, HH, HW, CW, CL, HI and HLN traits.


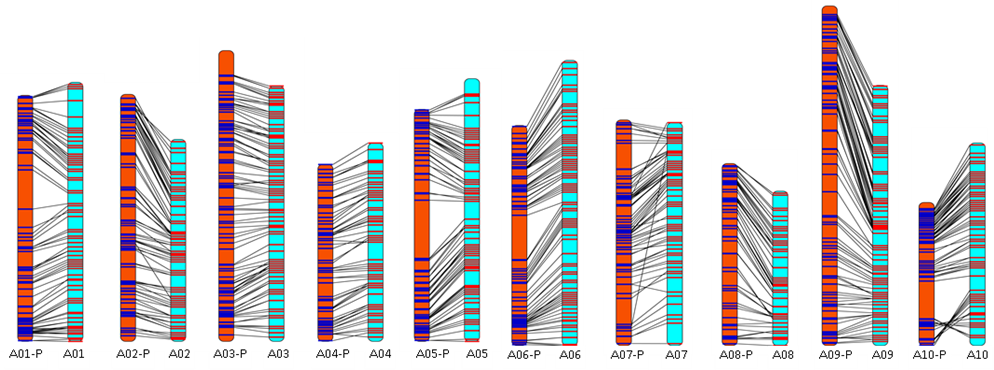


Figure S2. Genetic linkage map (orange) and Physical map (Chiifu reference genome: blue) of DH-88 consisting of 10 linkage groups with 1603 SNP markers.


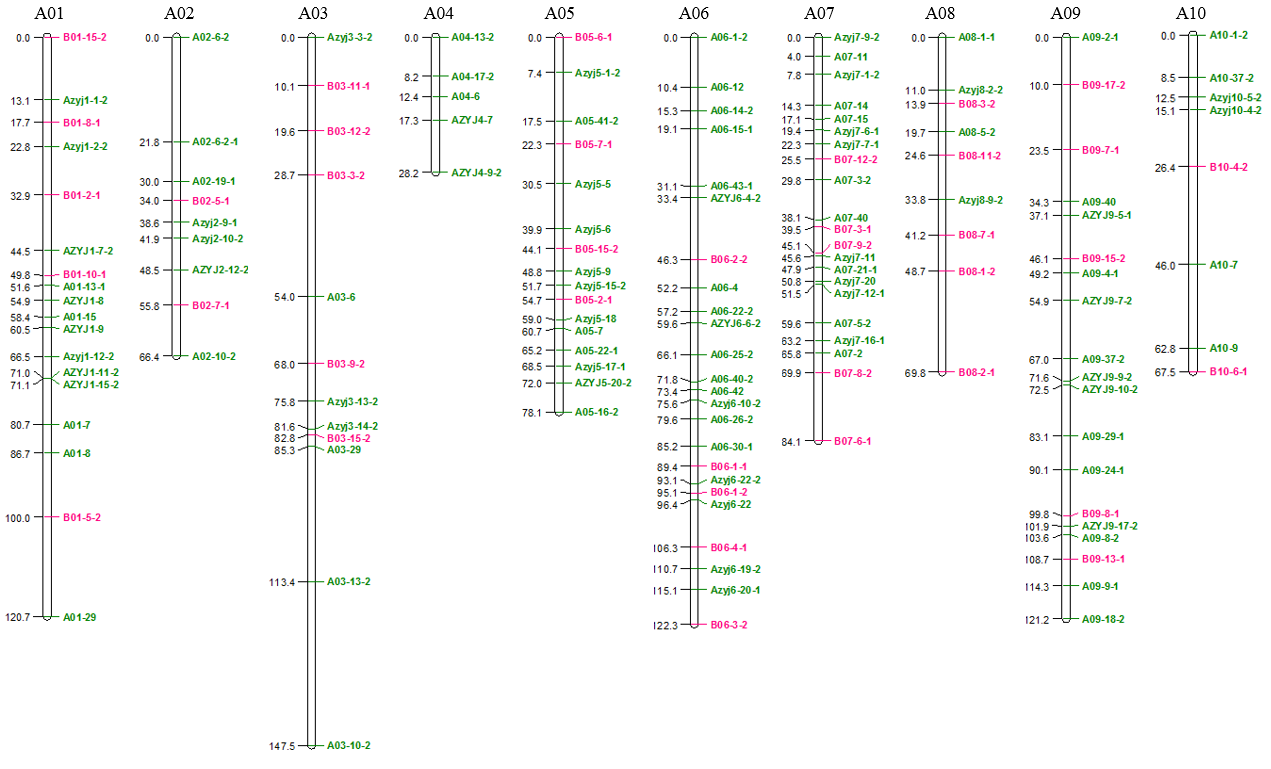


Figure S3. Genetic map of the F2-485 consisting of 10 linkage groups with 36 SNP and 99 InDel markers. Common markers between DH-88 and F2-485 are pink.

Table S4. The common SNP markers between DH-88 and F2-485 on A01 till A10

| **Common marker** | | **Linkage group** | **Primer number in F_2_ / DH-88** | **Common marker** | **Linkage group** | **Primer number in F_2_ / DH-88** |
| --- | --- | --- | --- | --- | --- | --- |
| S1a | | A01 | B01-15-2/ Bn-A01-p994722 | S7a | A07 | B07-12-2/ Bn-A07-p7027400 |
| S1b | | A01 | B01-10-1/ Bn-A01-p6376105 | S7b | A07 | B07-3-1/ Bn-A07-p10099786 |
| S1c | | A01 | B01-5-2/ Bn-A01-p24655110 | S7c | A07 | B07-8-2/ Bn-A07-p14958606 |
| S2a | | A02 | B02-2-1/ Bn-A02-p3422005 | S7d | A07 | B07-6-1/ Bn-A07-p13639374 |
| S2b | | A02 | B02-5-1/ Bn-A02-p19683093 | S7e | A07 | B07-9-2/ Bn-A07-p19977445 |
| S2c | | A02 | B02-7-1/ Bn-A02-p25181474 | S8a | A08 | B08-3-2/ Bn-A08-p4528943 |
| S3a | | A03 | B03-11-1/ Bn-A03-p5439400 | S8b | A08 | B08-11-2/ Bn-A08-p12761022 |
| S3b | | A03 | B03-12-2/ Bn-A03-p6919500 | S8c | A08 | B08-7-1/ Bn-A08-p14447288 |
| S3c | | A03 | B03-3-2/ Bn-A03-p8629992 | S8d | A08 | B08-1-2/ Bn-A08-p17586676 |
| S3d | | A03 | B03-9-2/ Bn-A03-p16812953 | S8e | A08 | B08-2-1/ Bn-A08-p20085504 |
| S3e | | A03 | B03-15-2/ Bn-A03-p20560569 | S9a | A09 | B09-17-2/ Bn-A09-p1243032 |
| S5a | | A05 | B05-6-1/ Bn-A05-p1671823 | S9b | A09 | B09-7-1/ Bn-A09-p2044817 |
| S5b | | A05 | B05-7-1/ Bn-A05-p2898061 | S9c | A09 | B09-15-2/ Bn-A09-p6632506 |
| S5c | | A05 | B05-15-2/ Bn-A05-p6337412 | S9d | A09 | B09-13-1/ Bn-A09-p30002747 |
| S5d | | A05 | B05-2-1/ Bn-A05-p18453140 | S9e | A09 | B09-8-1/ Bn-A09-p29348596 |
| S6a | | A06 | B06-2-2 /Bn-A06-p4452778 | S10a | A10 | B10-4-2/ Bn-A10-p9186877 |
| S6b | A06 | | B06-4-1/ Bn-A06-p22910339 | S10b | A10 | B10-6-1/ Bn-A10-p15031810 |

Table S5A. Summary statistics for the head traits in F2-485

|  | | | | | |
| --- | --- | --- | --- | --- | --- |
| Trait | PC-101 | CC-48 | Mean | S.D | Variance |
| HH | - | 52.4 | 34.57 | 5.081 | 25.81 |
| HW | - | 15.2 | 16.42 | 3.124 | 9.759 |
| HWe | - | 2.5 | 1.688 | 0.674 | 0.454 |

Table S5B. Correlation coefficients of head traits in F2-485

|  | | | |
| --- | --- | --- | --- |
| Trait | HW | HWe | HDe |
| HH | 0.214** | 0.434** | 0.118* |
| HW | 1 | 0.638** | 0.273** |
| HWe |  | 1 | 0.348** |

**Correlation is significant at the 0.01 level (2-tailed).

*Correlation is significant at the 0.05 level (2-tailed).

Note: HH= head high; HW= head width; Hwe= head weight; HDe= heading degree.
